# Field evaluation of newly developed 3D-printed ultraviolet and green light-emitting diode traps for the collection of *Culicoides* species in Thailand

**DOI:** 10.1371/journal.pone.0280673

**Published:** 2023-01-20

**Authors:** Yuki Fujisawa, Kandit Kornmatitsuk, Sudsaijai Kornmatitsuk, Bunlue Kornmatitsuk

**Affiliations:** 1 Department of Clinical Sciences and Public Health, Faculty of Veterinary Science, Mahidol University, Salaya, Phutthamonthon, Nakhon Pathom, Thailand; 2 Chulalongkorn University Demonstration Secondary School, Faculty of Education, Chulalongkorn University, Pathum Wan, Bangkok, Thailand; Onderstepoort Veterinary Institute, SOUTH AFRICA

## Abstract

*Culcioides* biting midges (Diptera: Ceratopogonidae) are vectors of various veterinary pathogens. Suction light traps are one of the most widely used tools for vector surveillance. The present aim was to compare the efficiency for the collection of *Culicoides* species between newly developed 3D-printed ultraviolet (Mahidol University (MU) UV LED) and green light-emitting diode (Mahidol University (MU) Green LED) traps baited with CO_2_ and UV LED Center for Disease Control (CDC) light trap (BioQuip 2770) baited with CO_2_. The experiment consisted of two replicates of a 3 × 3 Latin square design in each three sampling locations (Location 1, 2, 3 and 4, 5, 6), for 12 nights between 26th July and 7th August 2020 in Thailand. Results showed that efficiency of the MU UV LED light trap was equivalent to that of the BioQuip 2770 trap for the collection of *Culicoides*. Meanwhile, the efficiency of the MU Green LED light trap was lower than that of both UV LED light traps. In the analysis of *Culicoides* species composition and sex–age grading, a similar pattern was observed among three light traps except for *Culicoides actoni* Smith. The newly developed 3D-printed UV LED light trap demonstrated the following advantages over the commercial light trap: cost saving to obtain multiple units, ease of customization and standardization, and increased availability by end-users. Although further assessments in different environmental conditions are needed, this 3D-printed light trap design could minimize the constrains in vector surveillance programs worldwide.

## 1. Introduction

*Culicoides* biting midges (Diptera: Ceratopogonidae) are small hematophagous insects (1–3 mm in size) transmitting numerous veterinary pathogens. To date, more than 50 arboviruses have been isolated from a limited number of species of *Culicoides* midges [1]. In particular, the members of genus *Orbivirus* within family Reoviridae, such as bluetongue virus (BTV), epizootic hemorrhagic disease virus (EHDV), and African horse sickness virus (AHSV), have exerted a significantly negative effect on animal industries globally [2, 3]. As vector-borne diseases, their epidemiology is strongly correlated with vector distribution and relative abundance [4]. Hence, identifying the potential vector species is crucial for the development of disease control strategies.

Light traps are one of the primary monitoring tools used for *Culicoides* sampling. Similar to other phototactic Diptera, *Culicoides* species preferably respond to UV light compared with other light wavelengths [5]. Green light attracts certain species, including *C*. *brevitarsis* Kieffer [6, 7], *C*. *obsoletus* (Meigen), and *C*. *scoticus* Downes and Kettle [5]. Among a wide range of *Culicoides* light traps, the Onderstepoort Veterinary Institution (OVI) UV light-suction (UVLS) trap has been demonstrated as a sensitive, highly efficient tool for *Culicoides* surveillance [8]. However, its disadvantages are its 220 V main power requirements and limited commercial production [9]. Miniature CDC UVLS light trap (Model 1212) with a standard UV tube is commercially available and applied for *Culicoides* collection. This trap is powered by a rechargeable 6 V battery (2.2 kg) for use in the absence of main power, and its efficiency is equivalent or inferior to that of the OVI UVLS trap [8, 10].

Instead of standard UV bulbs or tubes, light-emitting diodes (LEDs) have been utilized in standard insect-vector traps [11]. The advantages of using LEDs are reduced power consumption compared with standard incandescent and UV light sources, accuracy in specific wavelength achievement, reduced operating temperatures, durability, compact size, and monochromatic light production in a wide variety of wavelengths (colors) [5, 9, 11, 12]. However, the LED trap has lower efficiency than the standard CDC UV trap probably as a result of the former’s lower lighting ability due to its smaller size [9]. Nevertheless, brightness level allows for flexibility in the electrical current, passing through the LED, LED bulbs, viewing angles, or light intensity. Specific lighting arrangements can be used to maximize either capture rates or battery life depending on the field application [11]. At present, the LED CDC light trap is commonly used for the collection of *Culicoides* [9].

In recent years, 3D printing become accessible and enabled the creation of various physical objects from digital modeling data using a 3D printer and various 3D printing materials, e.g., polylactic acid (PLA) and polyethylene terephthalate glycol (PETG). This technology has been applied to create a mosquito light trap whose efficiency for the collection of mosquitoes is comparable with that of commercially available CDC-light and BG-Sentinel 2 trap [13]. Although the average 3D printer setup requires an initial investment, the total cost is reduced when compared with purchasing the commercially available light traps [13].

In this study, we newly developed 3D-printed UV and green LED light traps named “Mahidol University (MU) UV LED light trap” and “MU Green LED light trap”, respectively. The efficiency of the light traps for the field collection of *Culicoides* were compared with that of a standard commercially available UV LED CDC trap (BioQuip 2770). This invention will simplify *Culicoides* collection and surveys, especially in areas where commercial light traps may not easily be accessible due to logistic problems. The traps may also provide an economical sampling tool for large-scale vector surveillance.

## 2. Materials and methods

The experimental protocol was reviewed and approved by the Mahidol University-Institute Animal Care and Use Committee (FVS-MU-IACUC, Protocol Number: MUVS-2020-02-06) in accordance with the ethical principles and guidelines for the use of animals by the National Research Council, Bangkok, Thailand.

### 2.1. Study area

The study was conducted at the Livestock and Wildlife Hospital, Faculty of Veterinary Science, Mahidol University, and the surrounding area in Sai Yok District, Kanchanaburi Province in western Thailand (Table 1). The study site is situated in a summer rainfall area, and the land is generally used for growing crops and rearing livestock. The sampling locations were selected based on *Culicoides* ecology and behavior: close to the areas where domestic ruminants are kept and close to the water and moist conditions, including canals, ponds, and mushes. Sampling locations were at least 20 m away from each other. Data of minimum and maximum temperature, and rainfall during 2020 were obtained from Kanchanaburi Meteorological Station located at about 50 km from the study site (S1 Fig).

**Table 1:** Description and collection data from six *Culicoides* sampling locations in Sai Yok District, Kanchanaburi Province, Thailand.

Location	Coordinates	Altitude (m)	Collection date	Farm animals (n)
1	14°08’39.5"N 99°08’41.3"E	210	26–31 Jul. 2020	23 ovines
2	14°08’07.9"N 99°09’03.5"E	239	26–31 Jul. 2020	20 caprines
3	14°08’08.5"N 99°09’04.6"E	238	26–31 Jul. 2020	3 bovines
4	14°08’39.7"N 99°08’40.8"E	209	2–7 Aug. 2020	10 bovines
5	14°08’54.6"N 99°08’40.7"E	206	2–7 Aug. 2020	41 caprines
6	14°08’09.1"N 99°08’53.7"E	238	2–7 Aug. 2020	11 bovines

### 2.2. Light trap comparisons

The *Culicoides* sampling efficiency of two newly developed 3D-printed LED light traps (MU UV LED and MU Green LED) baited with CO_2_ was compared with that of a standard commercial UV LED CDC light trap (BioQuip 2770, BioQuip Inc., USA) baited with CO_2_. The experiment consisted of two replicates of the 3 × 3 Latin square design in each of three sampling locations (Location 1, 2, 3 and 4, 5, 6), for 12 nights between 26th July and 7th August 2020 (Table 1). A single light trap, which had a thermos flask containing 1 kg of dry ice (sufficient to last the whole night) set above it, was employed at each sampling location. The spout of the flask was left open to facilitate the gradual flow of CO_2_ during the trap operation. The light trap was hung under the roof on a part of animal housing 1.5 m above the ground level. The battery was placed inside a waterproof bag for protection against rain. The traps were operated overnight from dusk (18:30) till dawn (06:30). The specimens were collected in 70% ethyl alcohol solution and kept at 4°C until further investigation.

### 2.3. Light trap design

**Fig 1:**
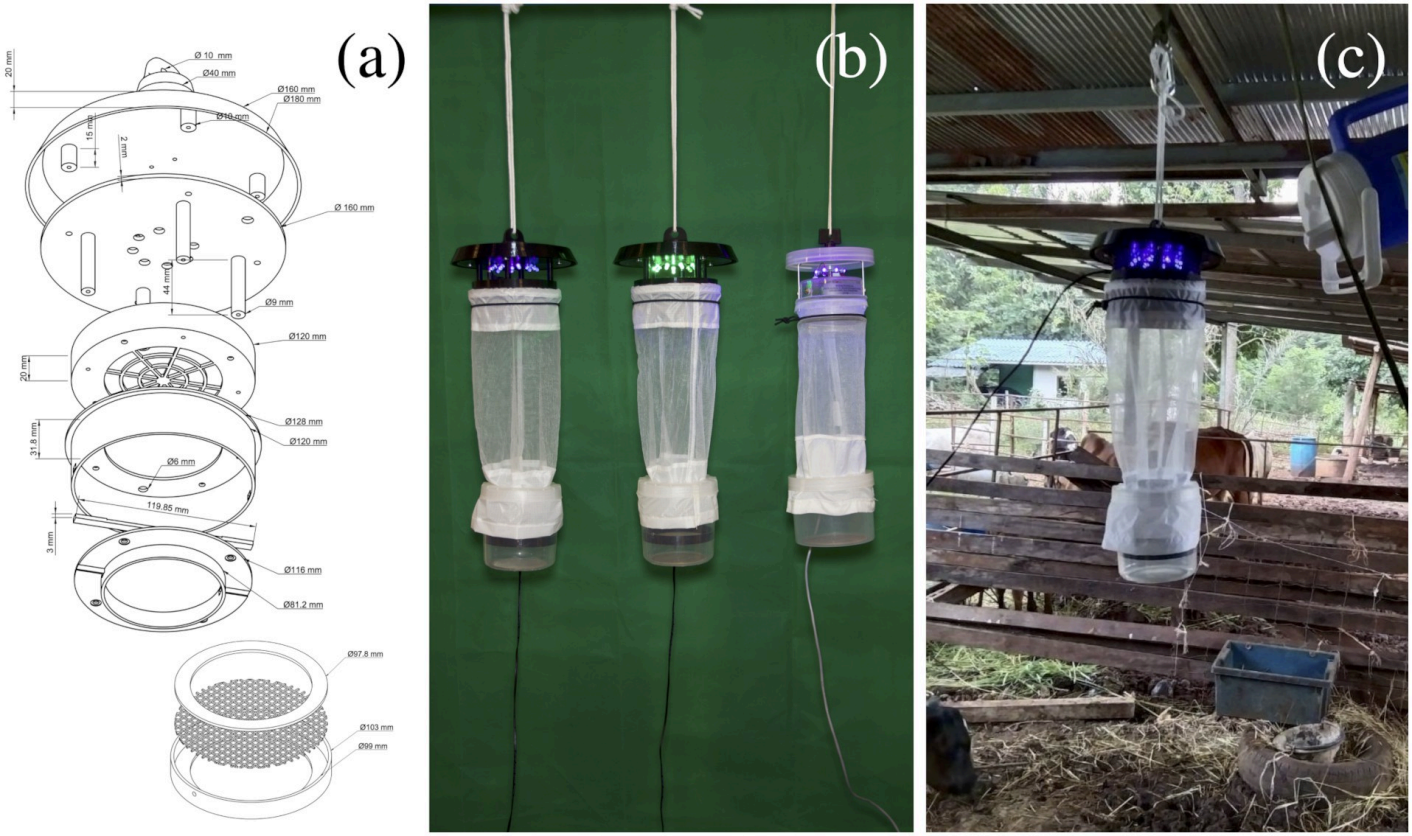
Light traps evaluated for the collection of *Culicoides*. (a) Newly designed 3D-printed LED light trap blueprint and a custom-designed *Culicoides* sorting-out frame (bottom); (b) trap size comparisons (left to right: MU UV LED, MU Green LED, and BioQuip 2770); and (c) MU UV LED light trap in field operation.

The 3D-printed LED light trap was designed using Fusion 360 software (Autodesk, Inc., CA, USA). A 3D printer (Ender 3 Pro, Shenzhen Creality 3D Technology Co., Ltd., Shenzhen, China) was employed to create the trap components using a black PETG filament (Fast Toner Co., Ltd., Bangkok, Thailand). The trap consisted of three main 3D-printed parts: 1) a body-part to house an 8 × 8 cm computer fan and a polyester screen (hole size: 5 mm) for preventing any materials or larger insects from entering the fan; 2) a light-part to house a printed circuit board and 8 UV or green LEDs (generic brand available from https://www.shopee.co.th); and 3) a rain shield-part for protecting the trap from rain and to facilitate the attachment of a string to hang the trap. All 3D-printed parts were assembled with metal screws. A 12V, 9 Ah battery was used to run the fan and LEDs. At the bottom of the body-part, a collection bag and cup (with metal nest #50, mesh size: 0.35 mm at the bottom) were attached. A custom-designed *Culicoides* sorting-out screen made from a 3D-printed PETG frame and metal nest #20 (mesh size: 0.96 mm) was incorporated within the collection cup (Fig 1).

### 2.4. *Culicoides* species identification

*Culicoides* specimens were separated from the other insects, identified, and counted. Each specimen was identified at the species level on the basis of the wing pattern and gross features using morphological keys under a stereomicroscope [14–16]. The specimens were then physiologically characterized by their abdomen: blood-fed, nulliparous (unpigmented), parous (pigmented), gravid females, and males [17]. Given that unpigmented and pigmented belonging to subgenus *Hoffmania* and *Trithecoides* are indistinguishable under the stereomicroscope, they were simply classified as non-blood-fed females. In collections containing more than 1,000 midges, a subsampling method [18] was used to estimate the abundance and diversity of *Culicoides* species.

#### 2.5. Statistical analysis

All data analyses were performed using the IBM SPSS Statistics for Windows, Version 23.0 (IBM Corp., Armonk, N.Y., USA). Count outcomes with the number of *Culicoides* collected per day among light traps were analyzed by generalized linear model (GLM) using Poisson regression model (PRM) and/or negative binomial regression model (NBRM), which is better than PRM in allowing the variance of the count response to be greater than the mean. Under PRM, *y*_*i*_ is the number of *Culicoides* with the average count *μ*_*i*_ where the exponential linking function links from the expected count *μ*_*i*_ to the predictor vector ***x***_***i***_ and the regression coefficient vector ***β***. With Poisson assumption, the variance of the count outcomes is equal to the mean ($\sigma_{i}^{2}=\mu_{i}$). Thus, under PRM:

$$y_{i}|x_{i}\;Poisson(\mu_{i}),\mu_{i}=exp(x_{i}\beta ),\sigma_{i}^{2}=\mu_{i}.$$

Alternatively, under NBRM:

$$y_{i}|x_{i}\;NegativeBinomial(y_{i}+\alpha^{-1},p_{i}),p_{i}=1/(1+\mu_{i}\alpha ),\mu_{i}=exp(x_{i}\beta ),\sigma_{i}^{2}=\mu_{i}+\alpha \mu_{i}^{2}.$$

The exponential link functions of PRM and NBRM are the same as *μ*_*i*_ = *exp*(***x***_***i***_***β***). However, the variance of NBRM with $\sigma_{i}^{2}=\mu_{i}+\alpha \mu_{i}^{2}$ is greater than that of PRM $\sigma_{i}^{2}=\mu_{i}$. If the dispersion parameter *α* equals 0, then NBRM variance is identical to the PRM variance.

The total *Culicoides* counts, *Culicoides* of different physiological stages counts, and relatively abundant *Culicoides* species counts were assessed. On the basis of data exploration and experimental design, the following model structure was used: the response variables were the total species count, species count in each physiological stage, and abundant species count per experimental day. The sampling location and date were modeled as random variables, and the type of traps (MU UV LED and MU Green LED light traps, and BioQuip 2770 light trap), which is the variable of interest for comparison, was modeled as an explanatory (fixed) variable. Meteorological data were assessed as random variables, but the date appeared to cover the variability introduced by these environmental variables. Akaike’s information criterion was applied for model selection, and statistical significance was set at *P* <0.05.

## 3. Results

### 3.1. Light trap design comparisons

The newly designed 3D-printed LED light trap was successfully produced using lightweight, durable, and temperature-resistant PETG materials. Excluding the battery and collection container, the trap weighed 335.8 g, which is approximately 122.7% of that of the commercial trap. The total cost of the 3D-printed LED light trap per unit including the initial capital investment (3D printer) was $221.69, which is $22.84 (or 11.5%) higher than that of the commercial product. However, the cost per 10 units revealed an 86.2% reduction ($275.42 for 10 units of MU UV LED or MU Green LED light trap and $1,988.50 for 10 units of BioQuip 2770 light trap). Specifications of 3D-printed LED light traps and standard UV LED light trap are shown in Table 2.

**Table 2:** Specifications of 3D-printed LED light traps (MU UV LED and MU Green LED) and standard UV LED light trap (BioQuip 2770) for the comparison of the efficiency for the collection of *Culicoides*.

	MU UV LED	MU Green LED	BioQuip 2770
Manufacturer	The end-user	The end-user	BioQuip Products, Inc. USA
Initial capital investment	3D printer, $215.72	3D printer, $215.72	$0
Light source	UV LED (8 LEDs); 0.16 Ah	Green LED (8 LEDs); 0.16 Ah	UV LED (8 LEDs).; 0.24 Ah
Wavelength	390–395 nm	525–535 nm	385–395 nm
Fan size; power consumption	80 mm; 0.14 Ah	80 mm; 0.14 Ah	70 mm; 0.11 Ah
Trap hour duration	~30 h	~30 h	~25 h
Rechargeable battery	12 V, 9 Ah lead-acid battery	12 V, 9 Ah lead-acid battery	6 V, 9 Ah lead-acid battery
Materials used^1^	PETG filament	PETG filament	Acrylic plastic
Collection container	Polypropylene (PP) cup and replaceable collection bag	Polypropylene (PP) cup and replaceable collection bag	Plastic cup and replaceable collection bag
Overall cost per 1 unit^2^	$221.69	$221.69	$198.85
Average overall cost for 10 units^2^	$275.42	$275.42	$1,988.50
Weight^3^	335.8 g	335.8 g	273.6 g
Other strengths	Lightweight, portable, and easily customizable. A *Culicoides* sorting-out screen is incorporated.		One of the smallest, lightweight, and most portable traps.

^1^ Only main body part

^2^ Prices are given in US dollar and import costs (transportation and tax) are not included

^3^ Excluding the collection bag, container, and battery

### 3.2. Total number and sex–age grading results of *Culicoides* specimens

A total of 96,334 *Culicoides* specimens were obtained in 36 collections for 12 nights. The MU UV LED light trap had the greatest capture (139.7% higher than that of BioQuip 2770 light trap) accounting for 51.4% of the total specimens collected. Significant differences in the mean numbers of total *Culicoides* collected were observed between the traps (X^2^ = 14.47, d.f. = 2, *P* = 0.001). The summary of *Culicoides* midges collected with three different light traps is shown in Table 3.

**Table 3:** Summary of *Culicoides* midges collected and overall sex–age grading results with three different LED light traps over 12 nights between 26th July and 7th August 2020 in Sai Yok District, Kanchanaburi Province, Thailand.

	MU UV LED	MU Green LED	BioQuip 2770	*Statistical significance
No. of collections	12	12	12	
No. of species	22	20	23	
Total number (%)	49,540 (51.4)^a^	11,322 (11.8)^b^	35,472 (36.8)^a^	X^2^ = 14.47, d.f. = 2, *P* = 0.001
Comparison with BioQuip 2770	139.7%	31.9%	100%	
Sex–age grading results				
Blood-fed (%)	2,797 (5.7)	996 (8.8)	3,291 (9.3)	X^2^ = 15.58, d.f. = 2, *P* <0.001
Nulliparous (%)	13,500 (27.3)	4,661 (41.2)	15,103 (42.6)	X^2^ = 25.06, d.f. = 2, *P* <0.001
Parous (%)	11,170 (22.6)	3,128 (27.6)	9,558 (28.0)	X^2^ = 16.87, d.f. = 2, *P* <0.001
Non-blood-fed (%)**	21,539 (43.5)	2,419 (21.4)	7,083 (20.0)	X^2^ = 28.08, d.f. = 2, *P* <0.001
Gravid (%)	294 (0.6)	79 (0.7)	304 (0.9)	X^2^ = 4.49, d.f. = 2, *P* = 0.106
Male (%)	240 (0.5)	39 (0.3)	133 (0.4)	X^2^ = 4.94, d.f. = 2, *P* = 0.091

* Indicates the statistical significance of trap type as a parameter in GLM

**This grading results came from only subgenus *Hoffmania* and *Trithecoides*.

**Fig 2:**
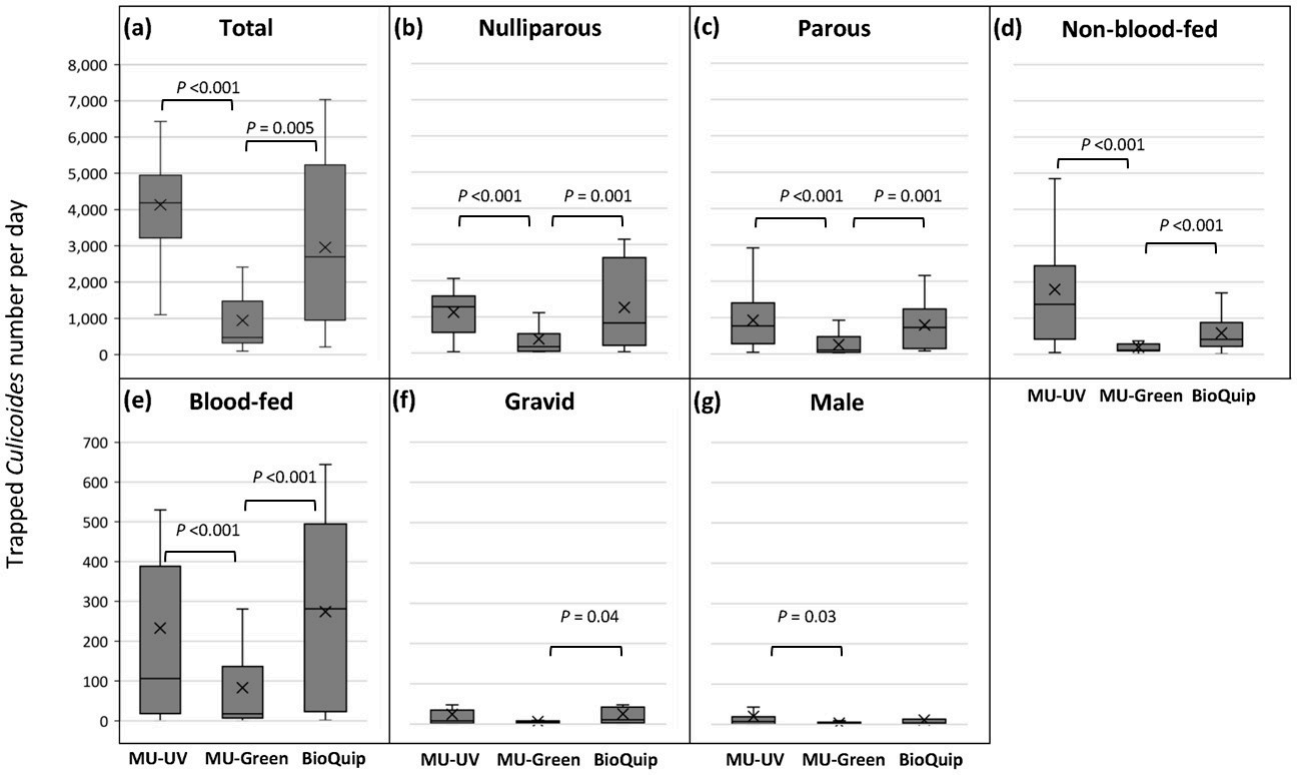
Box-and-whisker plots of daily number of *Culicoides* collected with three different LED light traps over 12 nights between 26^th^ July and 7^th^ August 2020 in Sai Yok District, Kanchanaburi Province, Thailand. The total count (a) and each physiological stage count (b–g) are shown. The cross marks in the middle of bars indicate the mean of the collection number per day.

The total number of *Culicoides* collected per day was not significantly different between MU UV LED and BioQuip 2770 light traps (*P* = 0.413). However, the total number of *Culicoides* collected per day was higher in MU UV LED and BioQuip 2770 light traps than in MU Green LED light trap by 4.38 times (*P <*0.001; 95% CI: 1.97–9.74) and 3.13 times (*P* = 0.005; 95% CI: 1.41–7.0), respectively (Fig 2a).

Nulliparous and parous, or non-blood-fed females comprised the majority of collected specimens with ranges of 27.3%–42.6%, 22.6%–27.6%, and 20.0%–43.5%, respectively. Blood-fed and gravid females and males were the minority groups with ranging of 5.7%–9.3%, 0.6%–0.9%, and 0.3%–0.5%, respectively. Similarly, sex–age grading results in the five most abundant *Culicoides* species were relatively consistent with the total counts of *Culicoides* collected (S1 Table).

The number of *Culicoides* in each physiological status collected with different traps were also compared, and significant difference were observed in blood-fed, nulliparous, parous, and non-blood-fed females. Similar to the total number, no statistically significant difference in collection size was found between MU UV LED and BioQuip 2770 light traps. Meanwhile, the collection sizes in MU UV LED and BioQuip 2770 light traps were larger than that in MU Green LED light trap as follows: nulliparous: 5.15 (*P* <0.001; 95% CI: 2.17–12.01) and 4.60 times (*P* = 0.001; 95% CI: 1.95–10.88), respectively (Fig 2b); parous: 5.20 (*P* <0.001; 95% CI: 2.25–12.01) and 3.94 times (*P* = 0.001; 95% CI: 1.70–9.14), respectively (Fig 2c); non-blood-fed: 9.13 (*P* <0.001; 95% CI: 3.95–21.10) and 5.09 times (*P* <0.001; 95% CI: 2.09–12.45), respectively (Fig 2d); blood-fed: 3.04 (*P* = 0.017; 95% CI: 1.22–7.61) and 6.37 times (*P* <0.001; 95% CI: 2.53–16.02), respectively (Fig 2e). Meanwhile, the collection size of gravid females in BioQuip 2770 was significantly larger by 2.82 times than in MU Green LED (*P* = 0.037; 95% CI: 1.07–7.45) (Fig 2f). The collection size of males in MU UV LED light trap was significantly larger by 2.90 times than in MU Green LED light trap (*P* = 0.029; 95% CI: 1.12–7.52) (Fig 2g). Although the numbers of various sex age grades differed significantly, the percentage representation of the different groups (at least majority and minority groups) collected with the various traps were comparable.

### 3.3. Number, positive catches, and rank of *Culicoides* species

Twenty-seven *Culicoides* species belonging to six subgenera (*Avaritia*, *Beltranmyia*, *Diphaomyia*, *Hoffmania*, *Remmia*, and *Trithecoides*) and three species groups (*Clavipalpis*, *Shermani*, and *Shortti*) including four unidentified species i.e., *Trithecoides* sp. 1, *Trithecoides* sp. 2, Unidentified sp. 1 and Unidentified sp. 2. *Trithecoides* sp. 1 and 2 were identified at the subgenus level according to the presence of three spermathecae [15]. The Unidentified sp. 1 and 2 had two spermathecae, which are found in most *Culicoides* genera. In addition, the BioQuip 2770 light trap was able to collect a female of *C*. *soleamaculatus* Nandi and Mazumdar (subgenus *Diphaomyia*), the first one recorded in Thailand (S2 Fig).

The most abundant species collected were *C*. *orientalis* Kieffer (56.4%; n = 54,318), followed by *C*. *innoxius* Sen and Das Gupta (25.8%; n = 24,892), *C*. *palpifer* Das Gupta and Ghosh (9.5%; n = 9,103), *C*. *jacobsoni* Macfie (4.8%; n = 4,660), and *C*. *actoni* (1.4%; n = 1,367). These five species composed more than 90% of all specimens collected in each light trap. Among the 27 species collected, 14 were present in all three different light traps as follows: *C*. *orientalis*, *C*. *innoxius*, *C*. *palpifer*, *C*. *jacobsoni*, *C*. *actoni*, *C*. *fulvus* Sen and Das Gupta, *C*. *oxystoma* Kieffer, *C*. *asiana* Bellis, *C*. *tainanus* Kieffer, *Trithecoides* sp. 1, *C*. *nigripes* Wirth and Hubert, *C*. *brevitarsis*, *C*. *huffi* Causey, and *C*. *shortti* Smith and Swaminath.

*Culicoides clavipalpis* Mukerji, *C*. *brevipalpis* Delfinado, *C*. *insignipennis* Macfie, *Trithecoides* sp. 2, *C*. *peregrinus* Kieffer, *C*. *imicola* Kieffer, *C*. *tenuipalpis* Wirth and Hubert, *C*. *soleamaculatus*, *C*. *dumdum* Sen and Das Gupta, *C*. *halonostictus* Wirth and Hubert, Unidentified sp. 1, Unidentified sp. 2, and *C*. *anophelis* Edwards were collected in low numbers in one or two of the three trap types. The summary of *Culicoides* species collected with three different light traps is shown in Table 4.

**Table 4:** Summary of *Culicoides* species collected with three different LED light traps over 12 nights between 26th July and 7th August 2020 in Sai Yok District, Kanchanaburi Province, Thailand.

Species	MU UV LED				MU Green LED				BioQuip 2770			
	Number	%	Positive catches	Rank	Number	%	Positive catches	Rank	Number	%	Positive catches	Rank
*C*. *orientalis*	23,471	47.4	12	1	7,813	69.0	12	1	23,034	64.9	12	1
*C*. *innoxius*	17,880	36.1	12	2	1,768	15.6	12	2	5,245	14.8	12	2
*C*. *palpifer*	5,239	10.6	12	3	1,013	8.9	12	3	2,851	8.0	12	3
*C*. *jacobsoni*	2,033	4.1	12	4	406	3.6	11	4	2,221	6.3	12	4
*C*. *actoni*	93	0.2	7	7	93	0.8	8	5	1,181	3.3	10	5
*C*. *fulvus*	266	0.5	10	5	52	0.5	8	7	335	0.9	11	6
*C*. *oxystoma*	252	0.5	10	6	65	0.6	9	6	187	0.5	10	7
*C*. *asiana*	77	0.2	6	9	16	0.1	6	9	115	0.3	7	8
*C*. *tainanus*	27	0.1	4	11	12	0.1	7	11	78	0.2	7	9
*Trithecoides* sp. 1	84	0.2	7	8	7	0.1	4	13	66	0.2	7	10
*C*. *nigripes*	41	0.1	6	10	34	0.3	7	8	66	0.2	6	10
*C*. *clavipalpis*	0	-	0	-	11	0.1	3	12	19	0.1	5	12
*C*. *brevitarsis*	16	< 0.05	3	12	1	< 0.05	1	17	17	< 0.05	4	13
*C*. *brevipalpis*	7	< 0.05	2	18	0	-	0	-	13	< 0.05	2	14
*C*. *huffi*	8	< 0.05	1	15	1	< 0.05	1	17	12	< 0.05	2	15
*C*. *insignipennis*	8	< 0.05	1	15	0	-	0	-	11	< 0.05	3	16
*Trithecoides* sp. 2	13	< 0.05	3	13	0	-	0	-	7	< 0.05	3	17
*C*. *shortti*	8	< 0.05	2	15	4	< 0.05	2	15	7	< 0.05	3	17
*C*. *peregrinus*	9	< 0.05	3	14	0	-	0	-	3	< 0.05	1	19
*C*. *imicola*	0	-	0	-	15	0.1	4	10	2	< 0.05	2	20
*C*. *tenuipalpis*	1	< 0.05	1	21	0	-	0	-	1	< 0.05	1	21
*C*. *soleamaculatus*	0	-	0	-	0	-	0	-	1	< 0.05	1	21
*C*. *dumdumi*	3	< 0.05	1	19	6	0.1	1	14	0	-	0	-
*C*. *halonostictus*	3	< 0.05	1	19	1	< 0.05	1	17	0	-	0	-
Unidentified sp. 1	1	< 0.05	1	21	0	-	0	-	0	-	0	-
Unidentified sp. 2	0	-	0	-	3	< 0.05	1	16	0	-	0	-
*C*. *anophelis*	0	-	0	-	1	< 0.05	1	17	0	-	0	-
**Total**	49,540				11,322				35,472			

### 3.4. Total number of five abundant *Culicoides* species

**Fig 3:**
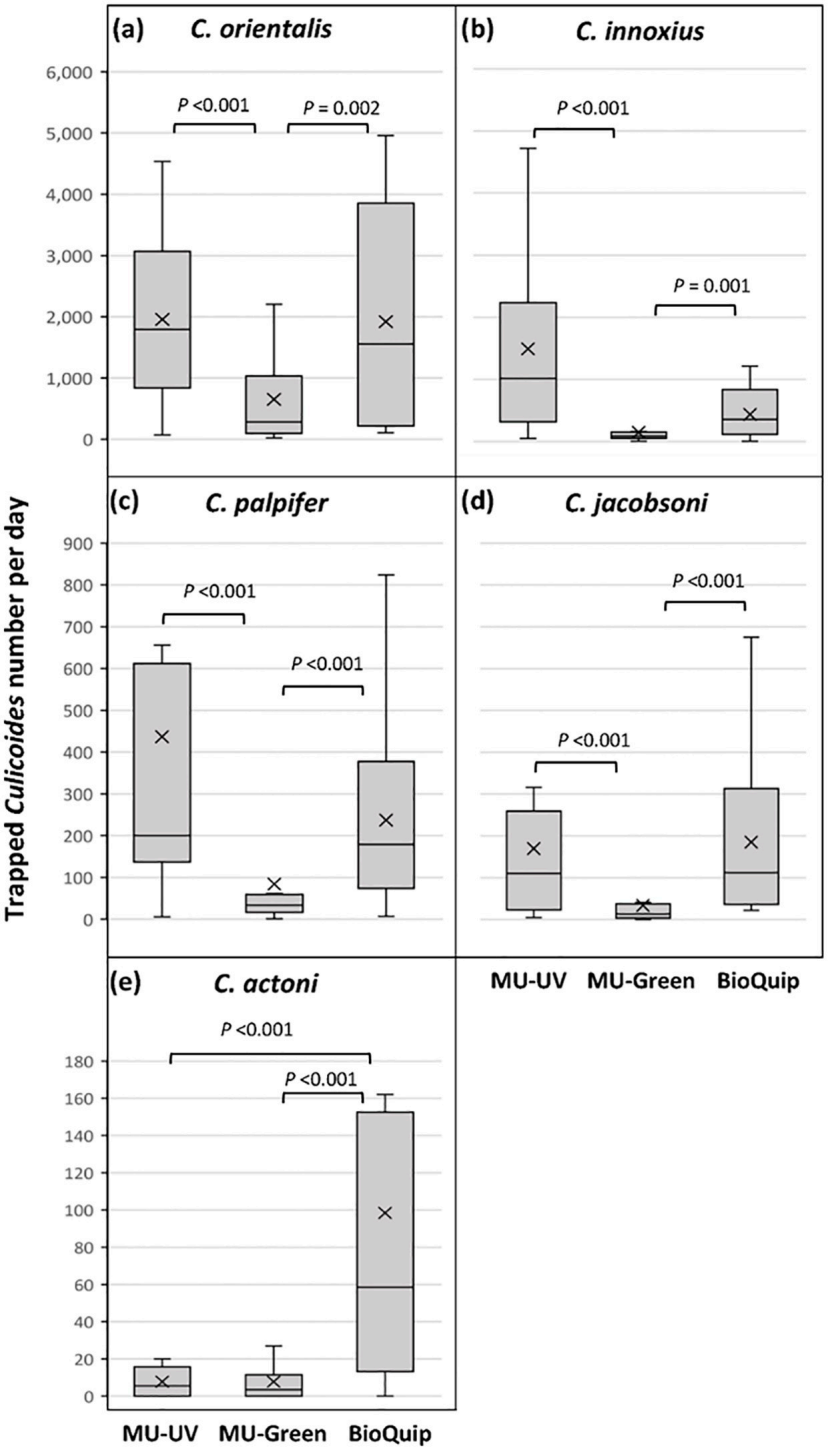
Box-and-whisker plots of the daily total number of the five most abundant *Culicoides* species collected with three different LED light traps over 12 nights between 26th July and 7th August 2020 in Sai Yok District, Kanchanaburi Province, Thailand. The total counts of the five most abundant species are shown. The cross marks in the middle of bars indicate the mean of the collection number per day.

The total numbers of the five most abundant *Culicoides* species, *C*. *orientalis*, *C*. *innoxius*, *C*. *palpifer*, *C*. *jacobsoni*, and *C*. *actoni* in each trap was compared. Except *C*. *actoni*, all these species showed the same pattern: No statistically significant difference in the number of the most abundant species collected was observed between MU UV LED and BioQuip 2770 light traps. The mean numbers of the most abundant *Culicoides* species collected in MU UV LED and BioQuip 2770 light traps were larger than that in MU Green LED light trap as follows: *C*. *orientalis*: 5.14 (*P* <0.001; 95% CI: 2.19–12.08) and 4.0 times (*P* = 0.002; 95% CI: 1.70–9.41), respectively (Fig 3a); *C*. *innoxius*: 9.44 (*P* <0.001; 95% CI: 4.06–21.96) and 5.02 times (*P* = 0.001; 95% CI: 2.01–12.53), respectively (Fig 3b); *C*. *palpifer*: 7.88 (*P* <0.001; 95% CI: 3.42–18.17) and 6.60 times (*P* <0.001; 95% CI: 2.76–15.80), respectively (Fig 3c) and *C*. *jacobsoni*: 6.60 (*P* <0.001; 95% CI: 2.61–16.28) and 9.62 times (*P* <0.001; 95% CI: 3.95–23.42), respectively (Fig 3d). The collection size of *C*. *actoni* was the largest in BioQuip 2770 light trap: 17.36 times larger than that in MU Green LED (*P* <0.001; 95% CI: 6.62–45.54) and 13.15 times larger than that in MU UV LED (*P* <0.001; 95% CI: 4.92–35.14). No statistically significant difference was found between MU UV LED and MU Green LED light traps (*P* = 0.588) (Fig 3e). The summary of sex–age grading results of the five most abundant *Culicoides* species collected with three different light traps is shown in S1 Table.

### 3.5. Effect from other factors

In the GLM, the location factor showed significant association and was added as parameter together with the total number of blood-fed (*P* <0.001), parous (*P* = 0.006), nulliparous (*P* = 0.002), non-blood-fed female (*P* = 0.025), and male (*P* <0.001) *Culicoides*. The number of gravid females was significantly affected by date more than type of traps (*P* = 0.001). Location significantly affected on the collection sizes of all the five abundant species as follows: *C*. *orientalis* (*P* = 0.001); *C*. *innoxius* (*P* = 0.060); *C*. *palpifer* (*P* <0.001); *C*. *jacobsoni* (*P* <0.001); *C*. *actoni* (*P* = 0.006).

## 4. Discussion

A newly designed 3D-printed LED light trap was successfully produced with total cost reduction of approximately 86.15% for 10 units compared with BioQuip 2770 light trap, despite the initial requirement of a 3D printer and printing accessories. This initial investment becomes cost effective and offers long-term cost savings once traps are produced for large-scale surveys [13]. In addition, the 3D-printed LED light trap reduces the cost of repairs, ensures standardization of the traps, allows for customization, and saves the shipping time and cost from aboard.

In this study, PETG was chosen as a manufacturing material for the 3D-printed LED light trap. The advantages of PETG over PLA, another common 3D printing material, include its high strength, and durability, very low shrinkage, temperature resistance, and excellent moisture blocking. These traits render this trap suitable to be deployed in the tropics with relative intense dew and rain. In addition, the custom-designed *Culicoides* sorting-out screen serves as an example of an innovative approach using 3D printing. The increasing availability of 3D printing facilitates the production of light traps by end-users, ensuring the rapid deployment of surveillance programs in case of unexpected disease outbreaks, such as the African horse sickness outbreak in Thailand in 2020 [19].

Although the total *Culicoides* collection sizes of MU UV LED and BioQuip 2770 light traps were statistically equivalent, the collection size in the BioQuip 2770 light trap was distributed with larger variability and approximately twice that in the MU UV LED light trap. In contrast, the MU Green LED light trap showed a smaller sampling size with narrower sample size distribution. The high efficiency of the MU Green LED light trap for the collection of certain *Culicoides* species was not observed in this study possibly due to the absence or low abundance of the species preferring green light over UV light, such as *C*. *brevitarsis*, *C*. *obsoletus* or *C*. *scoticus* [5–7].

Despite differences in the light source and trap bodies, the three light traps collected a consistent number of the most abundant species. In particular, *C*. *orientalis*, *C*. *innoxius*, *C*. *palpifer*, and *C*. *jacobsoni* accounted for more than 90% of all the *Culicoides* collected, making them the dominant *Culicoides* species at the study site. Some *Culicoides* species are either active during daylight hours when light traps are ineffective or poorly attracted to light traps [20, 21]. The collected single female of *C*. *soleamaculatus*, as identified by wing morphology, represented a new distribution record for this species in Thailand. This specimen represents the first collection of this specie outside India [16, 22]. Although this species comprises a trivial percentage of the total collection, the *Culicoides* influx from India is a possible cause of this finding [23]. Thus, the regular monitoring of regional *Culicoides* species distribution is crucial because vector-borne pathogens are expanding their range into new areas due to climate change and the presence of host and vector species [4, 24].

The sex–age structure of *Culicoides* collected showed a similar distribution among the three light traps. The same pattern was observed in the five most abundant species except in *C*. *actoni*, which preferred the BioQuip 2770 light trap more than the MU UV LED and MU Green LED light traps. Given that *C*. *actoni* is a diurnally active species, the light trap is a less effective sampling method for this insect [21]. Whether the present result is associated with the diurnal activity of *C*. *actoni* remains unclear. A possible reason is the difference in light source appearance (or output) between BioQuip 2770 and the 3D-printed LED light trap.

The sex–age structure of *Culicoides* populations is an important indicator associated with virus transmission. In this study, all the field samples were collected at ruminant farms, and all the traps showed a relatively high proportion of parous females. In the absence of transovarial virus transmission in *Culicoides*, only the parous females that have completed a gonotrophic cycle are able to transmit the virus via subsequent blood feeding [25, 26]. The species with a high parous rate should be remarked for further vector studies. However, pigmentation cannot be clearly observed, especially in members of certain subgenera; therefore, the reliability of their parous/nulliparous status identification is limited [27, 28].

All the light traps collected small proportions of males and females of blood-fed and gravid individuals in total and for the five most abundant species. This phenomenon could be the result of difference in the physiology of these groups. Given that the light traps were operated near the livestock animals, *Culicoides* females actively seeking blood meal were mainly collected, and males and only a few blood-fed and gravid females were collected because they were less attracted to livestock animals [8]. The proportional representation of *Culicoides* sexes depend on the distance from mating, breeding, and blood-feeding areas [29]; therefore, the sampling sites will be dictated by the goals and objectives of the study.

Finally, the use of light traps has negative impact on pathogen detection or transmission research as the apparent UV light aversion of BTV-infected *C*. *sonorensis* Wirth and Jones has been suggested [30, 31]. EHDV infection also has been associated with damage to some of the vision organs of *Culicoides* biting midges including ommatidia, optic ganglia, and Johnston’s organ, which could result in impaired function and subsequent behavioral changes of *C*. *sonorensis* [32]. Given the impact of virus infection on vector behavior, the reliance on UV light trapping for vectors may lead to the underestimation of transmission risk. However, this alteration in vector behavior in other *Culicoides* vector species or any specific viruses remains unclear and thus requires further investigation.

In conclusion, a newly designed 3D-printed UV LED light trap was successfully developed for the collection of *Culicoides* species that demonstrated several advantages over the commercial light trap BioQuip 2770: equivalent efficiency, cost saving for multiple units, ease of customization, and increased availability by end-users. A custom-designed 3D-printed sorting-out screen with nest #20 was also introduced for the effective removal of large non-target insects from light trap collections. Additionally, the proposed 3D-printed UV LED light trap design is applicable to a wide range of entomological surveillance.

## Supporting information

S1 Fig Monthly minimum and maximum temperature and amount of rainfall in the study area during 2020. (TIF)

S2 Fig *Culicoides soleamaculatus* wing photographed with a ×10 lens. Bar = 200 μm.
(TIF)

S1 Table Summary of the total number and sex–age grading results of the five most abundant *Culicoides* species collected with three different LED light traps over 12 nights between 26th July and 7th August 2020 in Sai Yok District, Kanchanaburi Province, Thailand. (DOCX)

S1 Text 3D-printed MU UV / MU Green LED light trap design. (PDF)
