# Supplementary figures and images for: Field evaluation of newly developed 3D-printed ultraviolet and green light-emitting diode traps for the collection of Culicoides species in Thailand
Source: PLoS One. 2023 Jan 20;18(1):e0280673. doi: 10.1371/journal.pone.0280673 (PMC9858794; doi:10.1371/journal.pone.0280673)

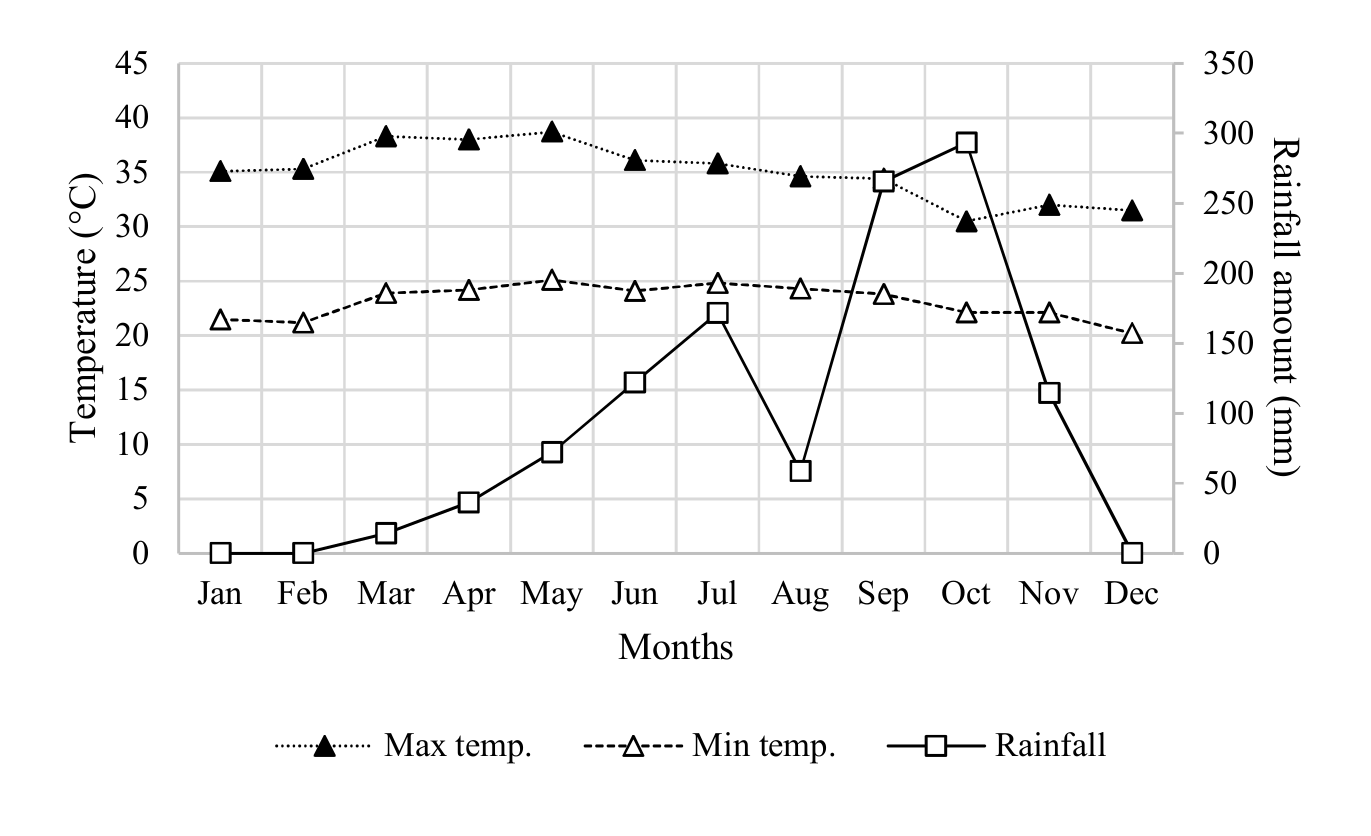

Supplement: S1 Fig — (TIF) [file pone.0280673.s001.tif]

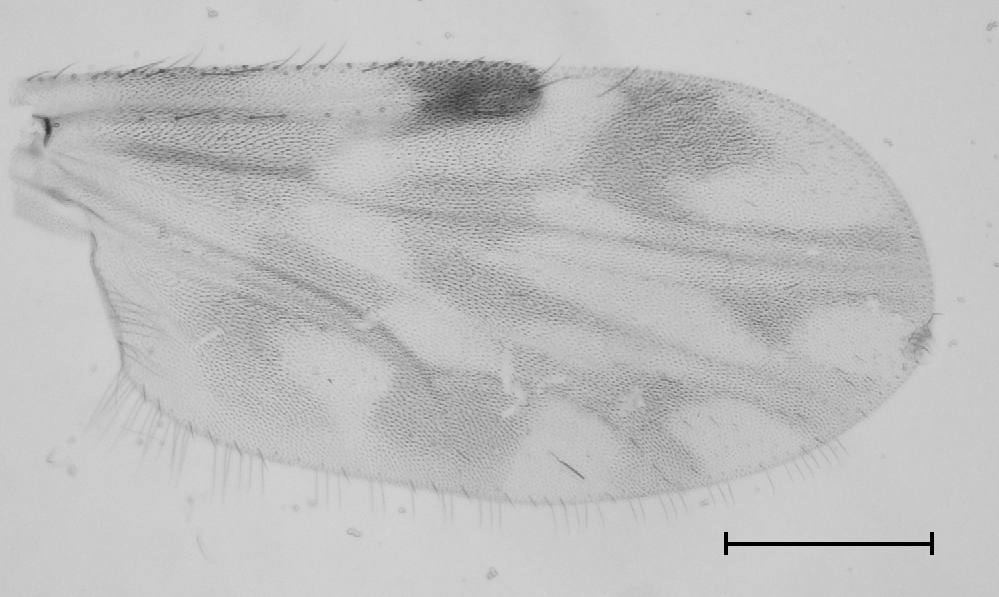

Supplement: S2 Fig — Bar = 200 μm. (TIF) [file pone.0280673.s002.tif]
