# Supplementary material for: Field evaluation of newly developed 3D-printed ultraviolet and green light-emitting diode traps for the collection of Culicoides species in Thailand
Source: PLoS One. 2023 Jan 20;18(1):e0280673. doi: 10.1371/journal.pone.0280673 (PMC9858794; doi:10.1371/journal.pone.0280673)
Supplement: S1 Table — (DOCX) [file pone.0280673.s003.docx]

**S1 Table.** **Summary of the total number and sex-age grading results of the five most abundant *Culicoides* species collected with three different LED light traps in three different LED traps over 12 nights between 26th July and 7th August 2020 in Sai Yok District, Kanchanaburi Province, Thailand.**

| **Species-sex/status** | **MU UV LED** | | **MU Green LED** | | **BioQuip 2770** | | ***Statistical significance** |  |
| --- | --- | --- | --- | --- | --- | --- | --- | --- |
| ***C. orientalis*** | **n (%)** | **mean ± SD** | **n (%)** | **mean ± SD** | **n (%)** | **mean ± SD** |  | |
| Total | 23,471 | 1,955.92 ± 1,331.92 | 7,813 | 651.08 ± 767.63 | 23,034 | 1,919.50 ± 1,793.22 | X^2^ = 16.11, d.f. = 2, *P* <0.001 | |
| Blood-fed | 1,199 (5.1) | 99.92 ± 123.46 | 450 (5.8) | 37.50 ± 49.94 | 1,764 (7.7) | 147.00 ± 135.11 | X^2^ = 5.14, d.f. = 2, *P* = 0.077 | |
| Nulliparous | 12,285 (52.3) | 1,023.75 ± 622.56 | 4,462 (57.1) | 371.83 ± 486.76 | 13,312 (57.8) | 1,109.33 ± 1,149.50 | X^2^ = 15.82, d.f. = 2, *P* <0.001 | |
| Parous | 9,931 (42.3) | 827.58 ± 708.55 | 2,878 (36.8) | 239.83 ± 288.53 | 7,906 (34.3) | 658.83 ± 635.52 | X^2^ = 16.79, d.f. = 2, *P* <0.001 | |
| Gravid | 41 (0.17) | 3.42 ± 7.03 | 16 (0.20) | 1.33 ± 1.92 | 48 (0.21) | 4.00 ± 7.15 | n/a | |
| Male | 15 (0.1) | 1.25 ± 2.05 | 7 (0.1) | 0.58 ± 1.16 | 4 (0.0) | 0.33 ± 1.15 | n/a | |
| ***C. innoxius*** |  |  |  |  |  |  |  | |
| Total | 17,880 | 1,490.00 ± 1,541.66 | 1,768 | 147.33 ± 169.50 | 5,245 | 437.08 ± 408.52 | X^2^ = 28.25, d.f. = 2, *P* <0.001 | |
| Blood-fed | 1,257 (7.0) | 104.75 ± 159.76 | 218 (12.3) | 18.17 ± 35.25 | 623 (11.9) | 51.92 ± 57.12 | X^2^ = 15.89, d.f. = 2, *P* <0.001 | |
| Non-blood-fed | 16,413 (91.8) | 1,367.75 ± 1,449.93 | 1,516 (85.8) | 126.33 ± 143.64 | 4,424 (84.3) | 368.67 ± 348.16 | X^2^ = 31.70, d.f. = 2, *P* <0.001 | |
| Gravid | 65 (0.36) | 5.42 ± 13.71 | 29 (1.6) | 2.42 ± 3.85 | 120 (2.3) | 10.00 ± 19.64 | n/a | |
| Male | 145 (0.81) | 12.08 ± 33.12 | 5 (0.28) | 0.42 ± 1.16 | 78 (1.5) | 6.50 ± 15.66 | n/a | |
| ***C. palpifer*** |  |  |  |  |  |  |  | |
| Total | 5,239 | 436.58 ± 470.28 | 1,013 | 84.42 ± 134.03 | 2,851 | 237.58 ± 239.90 | X^2^ = 27.38, d.f. = 2, *P* <0.001 | |
| Blood-fed | 131 (2.5) | 10.92 ± 21.12 | 115 (11.4) | 9.58 ± 22.22 | 222 (7.8) | 18.50 ± 30.57 | n/a | |
| Non-blood-fed | 5,041 (96.2) | 420.08 ± 444.92 | 895 (88.4) | 74.58 ± 113.60 | 2,595 (91.0) | 216.25 ± 217.79 | X^2^ = 21.93, d.f. = 2, *P* <0.001 | |
| Gravid | 63 (1.2) | 5.25 ± 11.41 | 2 (0.2) | 0.17 ± 0.39 | 33 (1.2) | 2.75 ± 4.59 | n/a | |
| Male | 4 (0.1) | 0.33 ± 1.15 | 1 (0.1) | 0.08 ± 02.9 | 1 (0.0) | 0.08 ± 0.29 | n/a | |
| ***C. jacobsoni*** |  |  |  |  |  |  |  | |
| Total | 2,033 | 169.42 ± 184.94 | 406 | 33.83 ± 51.44 | 2,221 | 185.08 ± 202.90 | X^2^ = 27.60, d.f. = 2, *P* <0.001 | |
| Blood-fed | 164 (8.1) | 13.67 ± 19.27 | 174 (42.9) | 14.50 ± 30.69 | 417 (18.8) | 34.75 ± 32.85 | X^2^ = 24.12, d.f. = 2, *P* <0.001 | |
| Nulliparous | 849 (41.8) | 70.75 ± 65.67 | 92 (22.7) | 7.67 ± 8.54 | 841 (37.9) | 70.08 ± 84.29 | X^2^ = 26.60, d.f. = 2, *P* <0.001 | |
| Parous | 934 (45.9) | 77.83 ± 114.61 | 132 (32.5) | 11.00 ± 13.99 | 937 (42.2) | 78.08 ± 92.99 | X^2^ = 26.46, d.f. = 2, *P* <0.001 | |
| Gravid | 44 (2.2) | 3.67 ± 11.50 | 6 (1.5) | 0.50 ± 1.24 | 8 (0.4) | 0.67 ± 1.56 | n/a | |
| Male | 42 (2.1) | 3.50 ± 8.73 | 2 (0.5) | 0.17 ± 0.39 | 18 (0.8) | 1.50 ± 2.61 | n/a | |
| ***C. actoni*** |  |  |  |  |  |  |  | |
| Total | 93 | 7.75 ± 8.35 | 93 | 7.75 ± 10.89 | 1,181 | 98.42 ± 128.43 | X^2^ = 41.18, d.f. = 2, *P* <0.001 | |
| Blood-fed | 8 (8.6) | 0.67 ± 1.56 | 20 (21.5) | 1.67 ± 3.42 | 187 (15.8) | 15.58 ± 19.81 | n/a | |
| Nulliparous | 25 (26.9) | 2.08 ± 5.82 | 22 (23.7) | 1.83 ± 3.04 | 500 (42.3) | 41.67 ± 64.01 | n/a | |
| Parous | 60 (64.5) | 5.00 ± 6.48 | 47 (50.5) | 3.92 ± 5.00 | 468 (39.6) | 39.00 ± 58.21 | n/a | |
| Gravid | 0 | - | 4 (4.3) | 0.33 ± 0.78 | 24 (2.0) | 2.00 ± 4.65 | n/a | |
| Male (%) | 0 | - | 0 | - | 2 (0.2) | 0.17 ± 0.39 | n/a | |
| **Five most abundant species** |  |  |  |  |  |  |  | |
| Total | 48,716 | 4,059.67 ± 2,326.12 | 11,093 | 924.42 ± 841.18 | 34,532 | 2,877.67 ± 2,362.32 | X^2^ = 14.38, d.f. = 2, *P* = 0.001 | |
| Blood-fed | 2,759 (5.7) | 229.92 ± 301.72 | 977 (8.8) | 81.42 ± 128.26 | 3,213 (9.3) | 267.75 ± 235.27 | X^2^ = 8.67, d.f. = 2, *P* = 0.013 | |
| Nulliparous | 13,159 (27.0) | 1,096.58 ± 641.58 | 4,576 (41.3) | 381.33 ± 484.93 | 14,653 (42.4) | 1,221.08 ± 1,178.65 | X^2^ = 16.80, d.f. = 2, *P* <0.001 | |
| Parous | 10,925 (22.4) | 910.42 ± 787.67 | 3,057 (27.6) | 254.75 ± 288.98 | 9,311 (27.0) | 775.92 ± 674.31 | X^2^ = 17.17, d.f. = 2, *P* <0.001 | |
| Non-blood-fed | 21,454 (44.0) | 1,787.83 ± 1,635.03 | 2,411 (21.7) | 200.92 ± 232.72 | 7,019 (20.3) | 584.92 ± 538.98 | X^2^ = 28.04, d.f. = 2, *P* <0.001 | |
| Gravid | 213 (0.4) | 17.75 ± 36.28 | 57 (0.5) | 4.75 ± 6.44 | 233 (0.7) | 19.42 ± 27.23 | X^2^ = 6.781, d.f. = 2, *P* = 0.034 | |
| Male | 206 (0.4) | 17.17 ± 37.84 | 15 (0.1) | 1.25 ± 1.36 | 103 (0.3) | 8.58 ± 16.68 | X^2^ = 9.87, d.f. = 2, *P* = 0.007 | |

* Indicates the statistical significance of trap type as a parameter in GLMs, (*P* <0.05).

n/a = Sample size was not enough in GLMs.
