# Supplementary material for: Field evaluation of newly developed 3D-printed ultraviolet and green light-emitting diode traps for the collection of Culicoides species in Thailand
Source: PLoS One. 2023 Jan 20;18(1):e0280673. doi: 10.1371/journal.pone.0280673 (PMC9858794; doi:10.1371/journal.pone.0280673)
Supplement: S1 Text — (PDF) [file pone.0280673.s004.pdf]

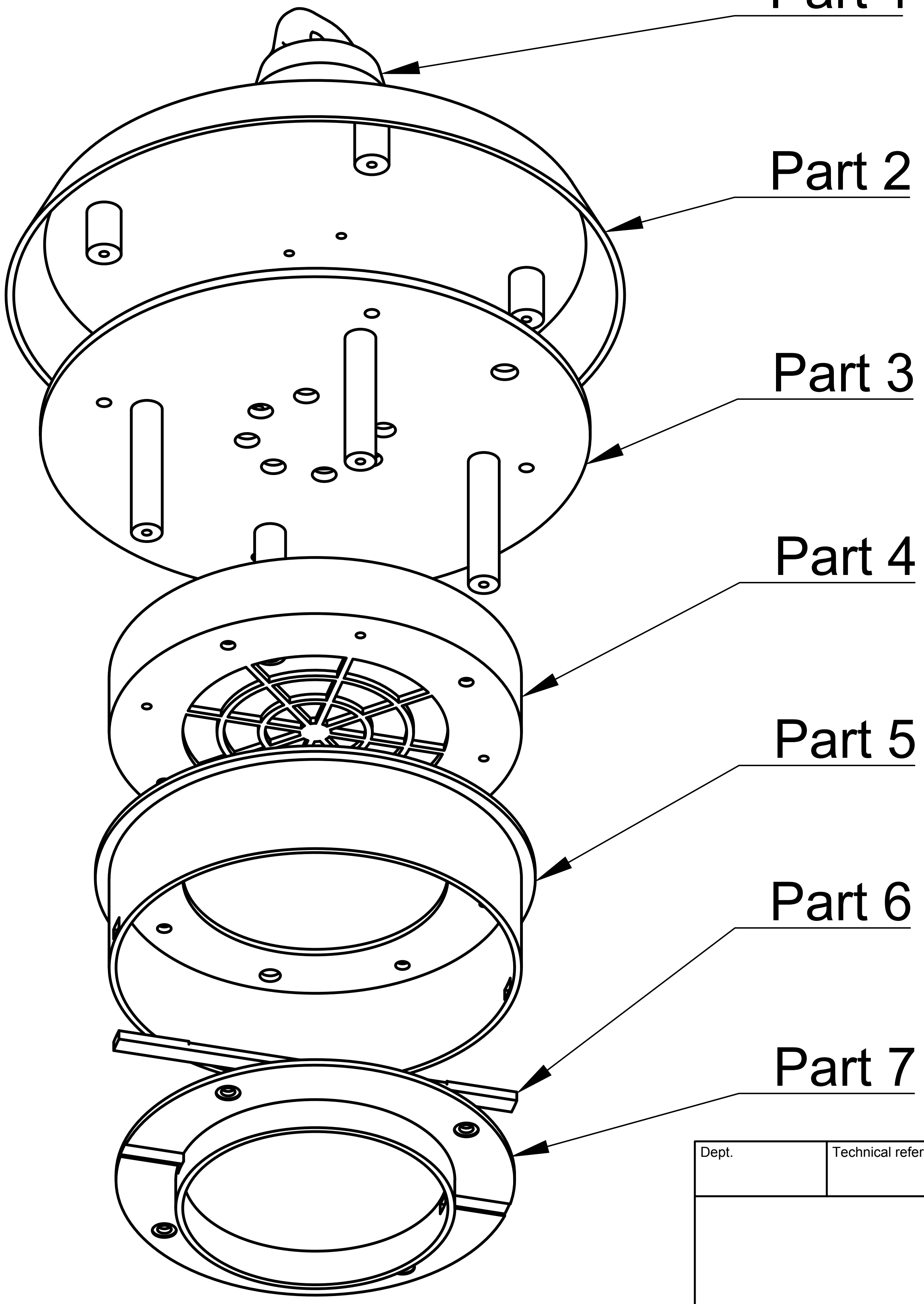

Dept.	Technical reference	Created by<br>Bunlue Kornmatitsuk and Kandit Kornmatitsuk	Approved by<br>Bunlue Kornmatitsuk and Kandit Kornmatitsuk
		Document type	Document status
		Title<br>MU UV/Green LED light trap	DWG No.
			Rev. / Date of issue / Sheet

Top view

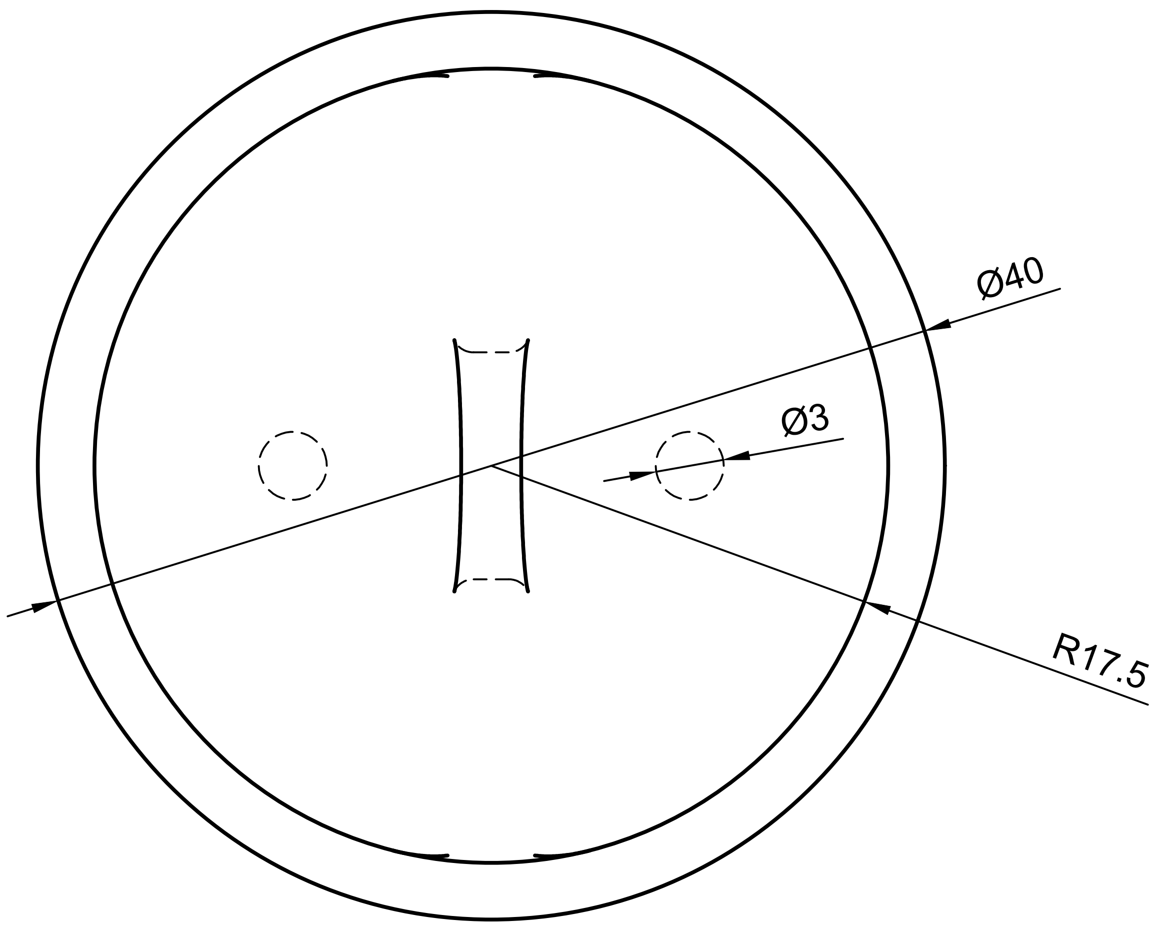

Perspective

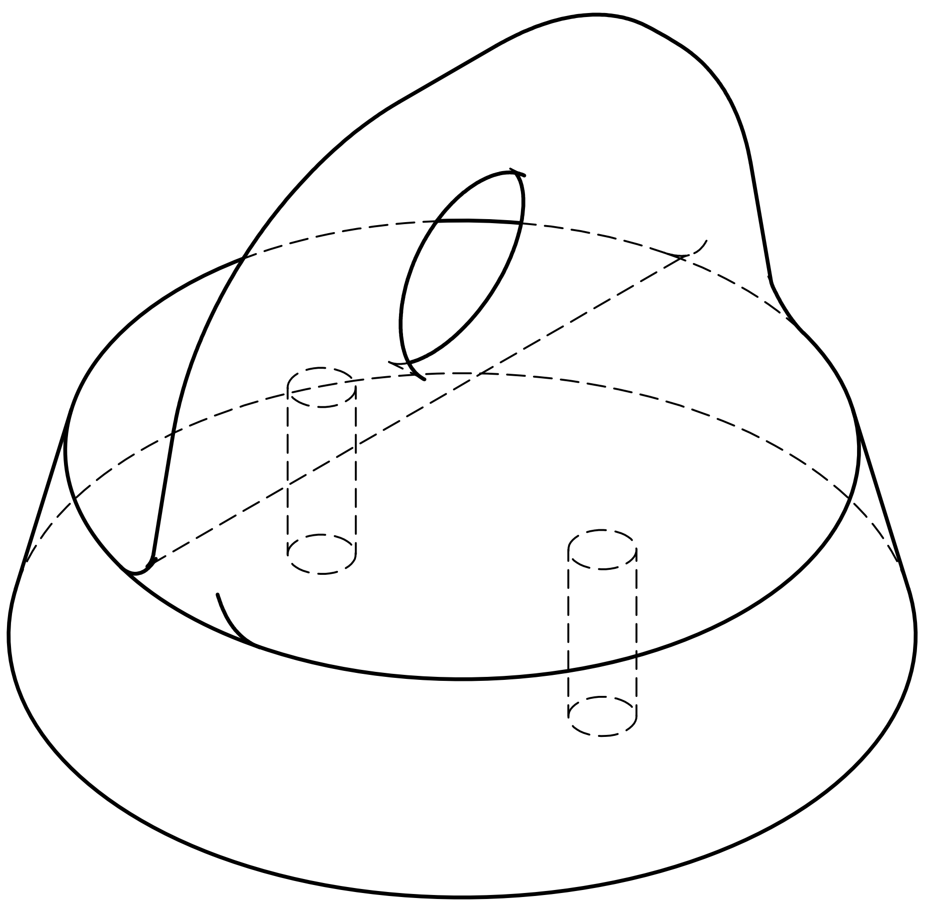

Front view

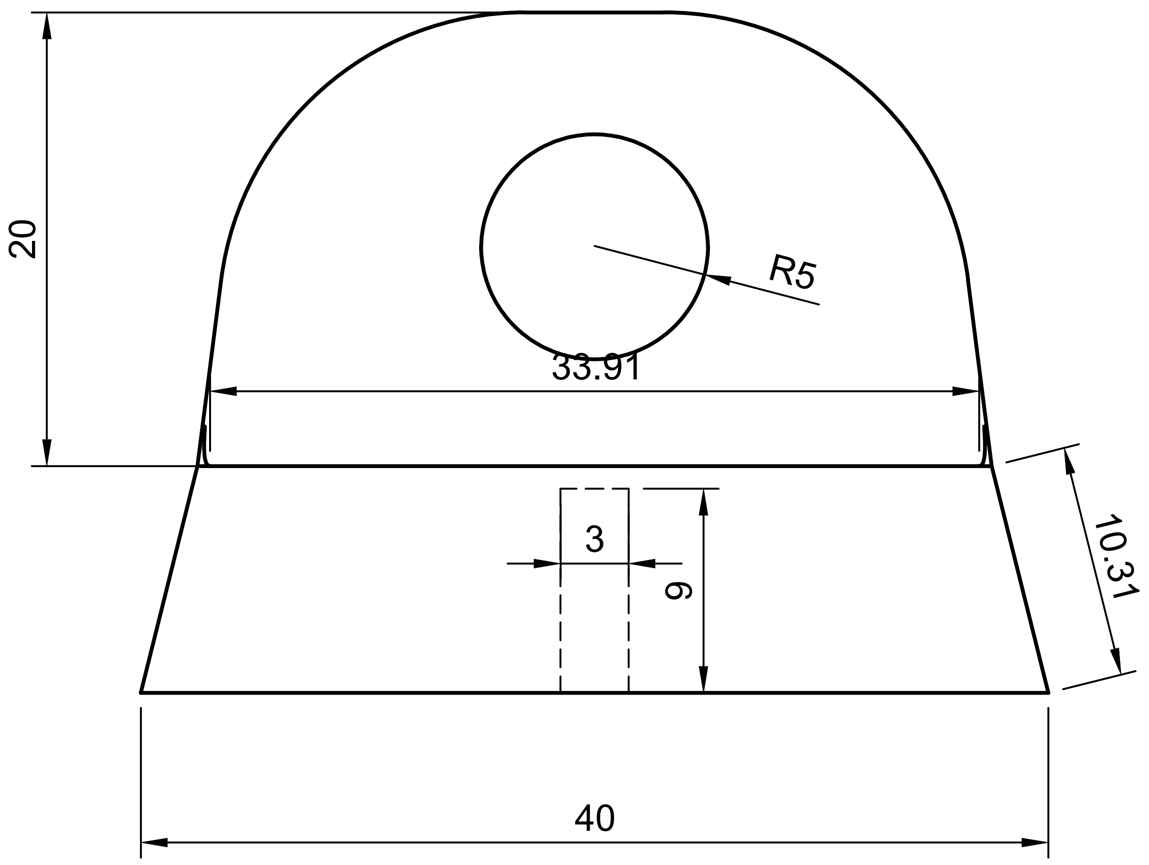

Right view

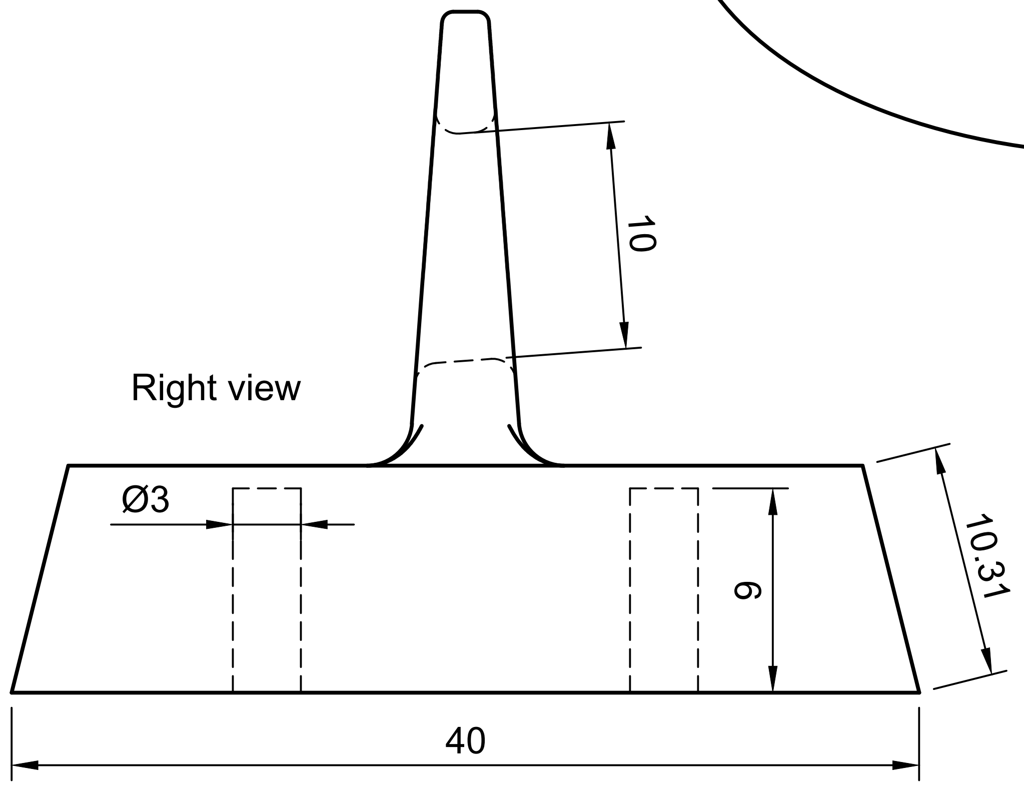

Dept.	Technical reference	Created by<br>Bunlue Kornmatitsuk and Kandit Kornmatitsuk	Approved by<br>Bunlue Kornmatitsuk and Kandit Kornmatitsuk		
		Document type	Document status		
		Title<br>MU UV/Green LED light trap<br>Part 1	DWG No.		
			Rev.	Date of issue	Sheet

Front View

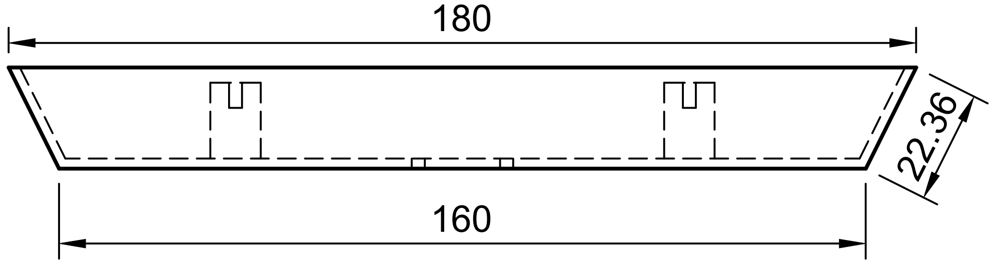

Top view

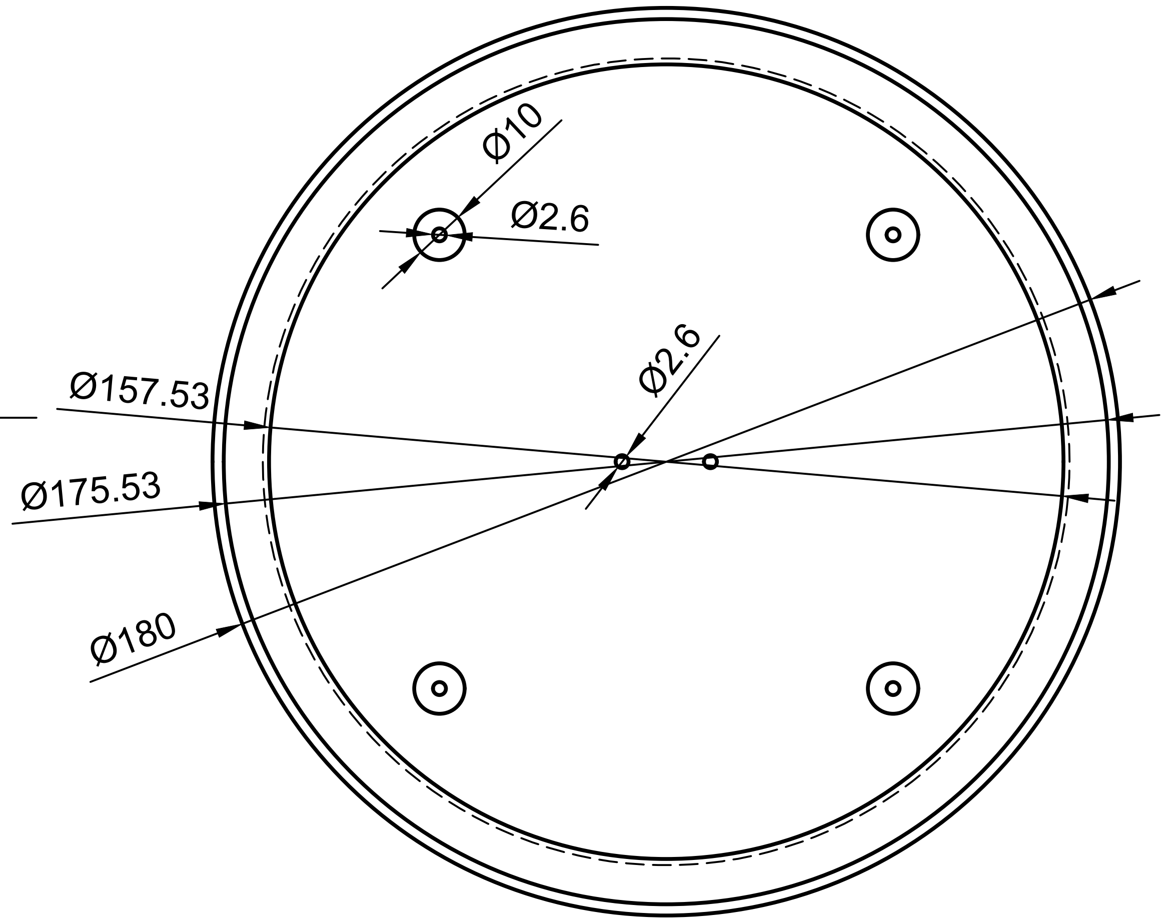

Perspective

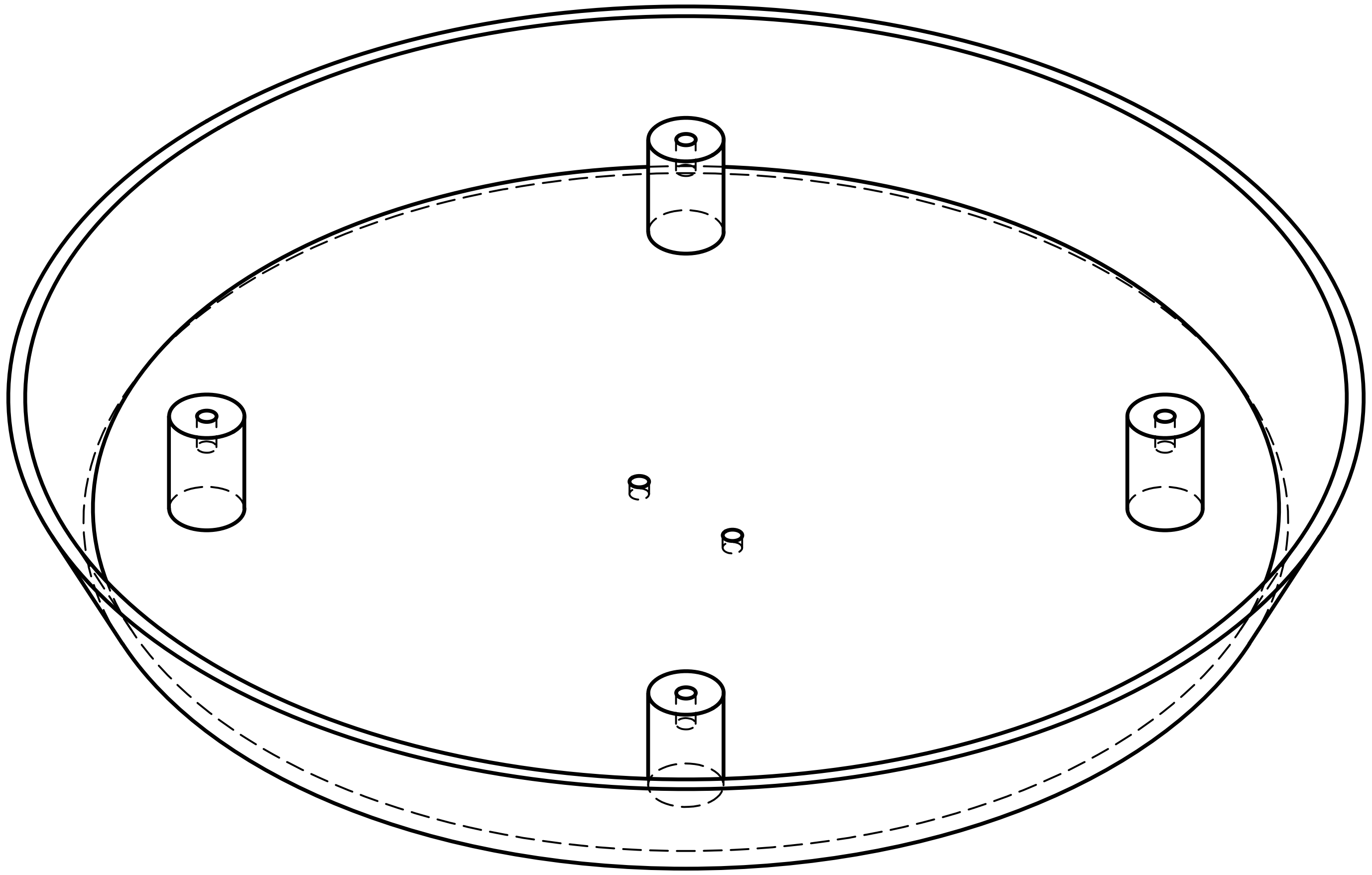

Dept.	Technical reference	Created by<br>Bunlue Kornmatitsuk and Kandit Kornmatitsuk	Approved by<br>Bunlue Kornmatitsuk and Kandit Kornmatitsuk
		Document type	Document status
		Title<br>MU UV/Green LED light trap<br>Part 2	DWG No.
			Rev. / Date of issue / Sheet

Top View

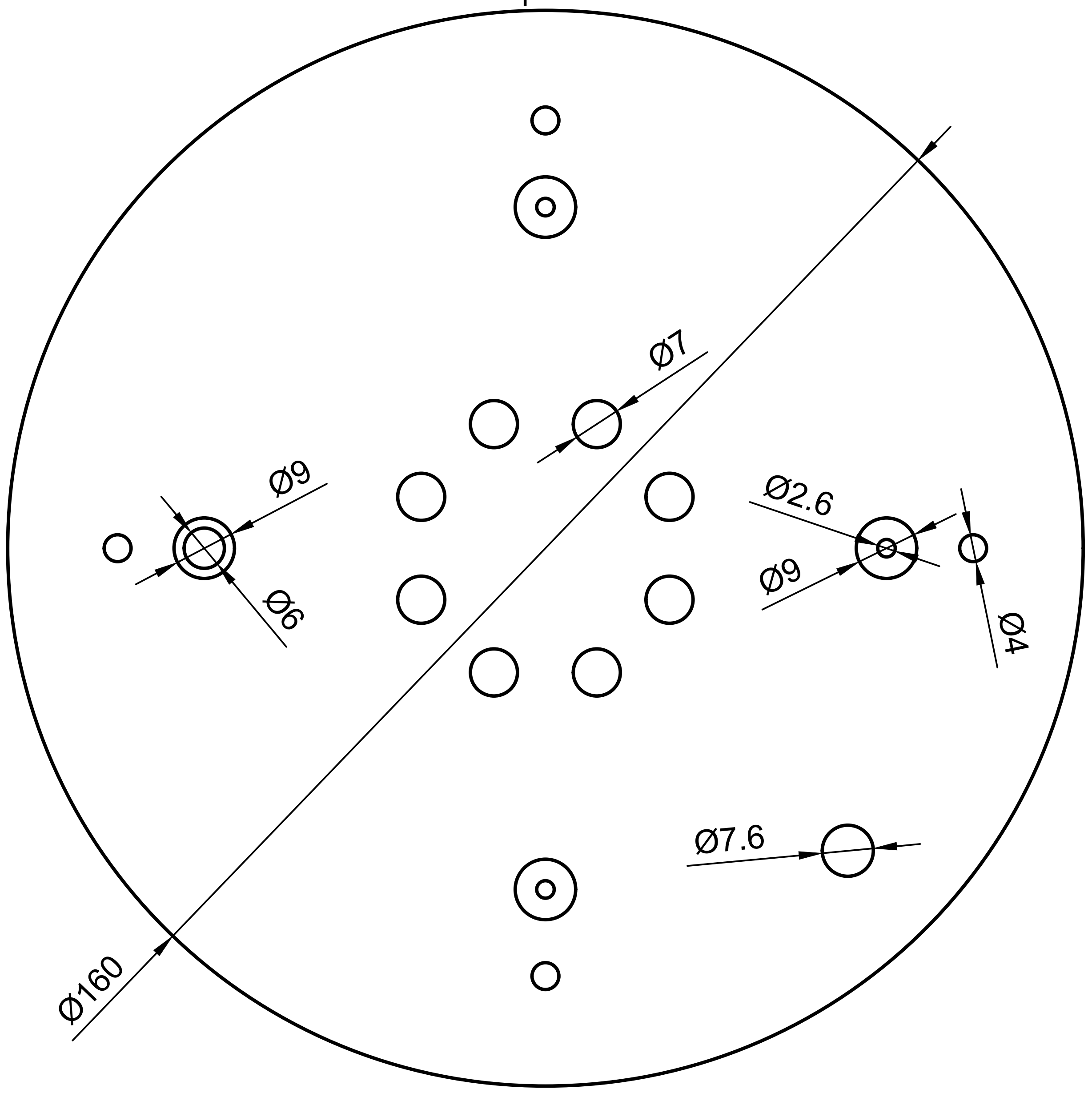

Front view

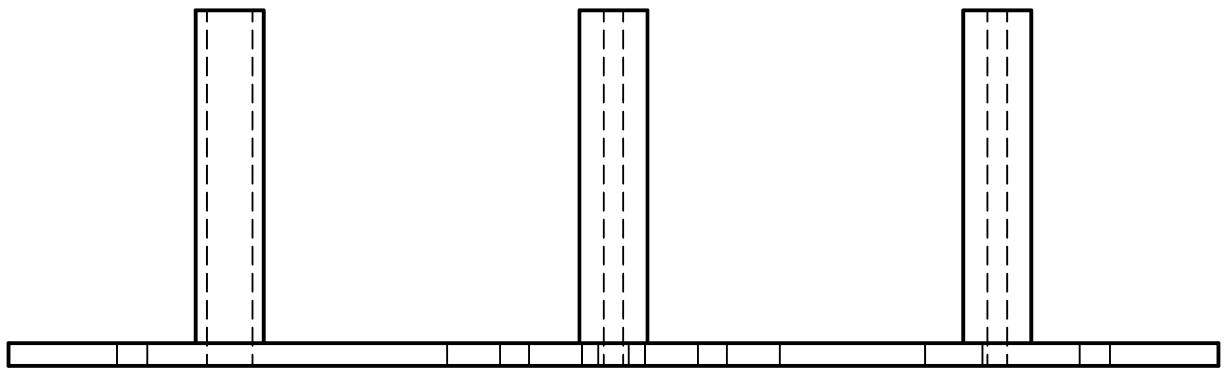

Perspective

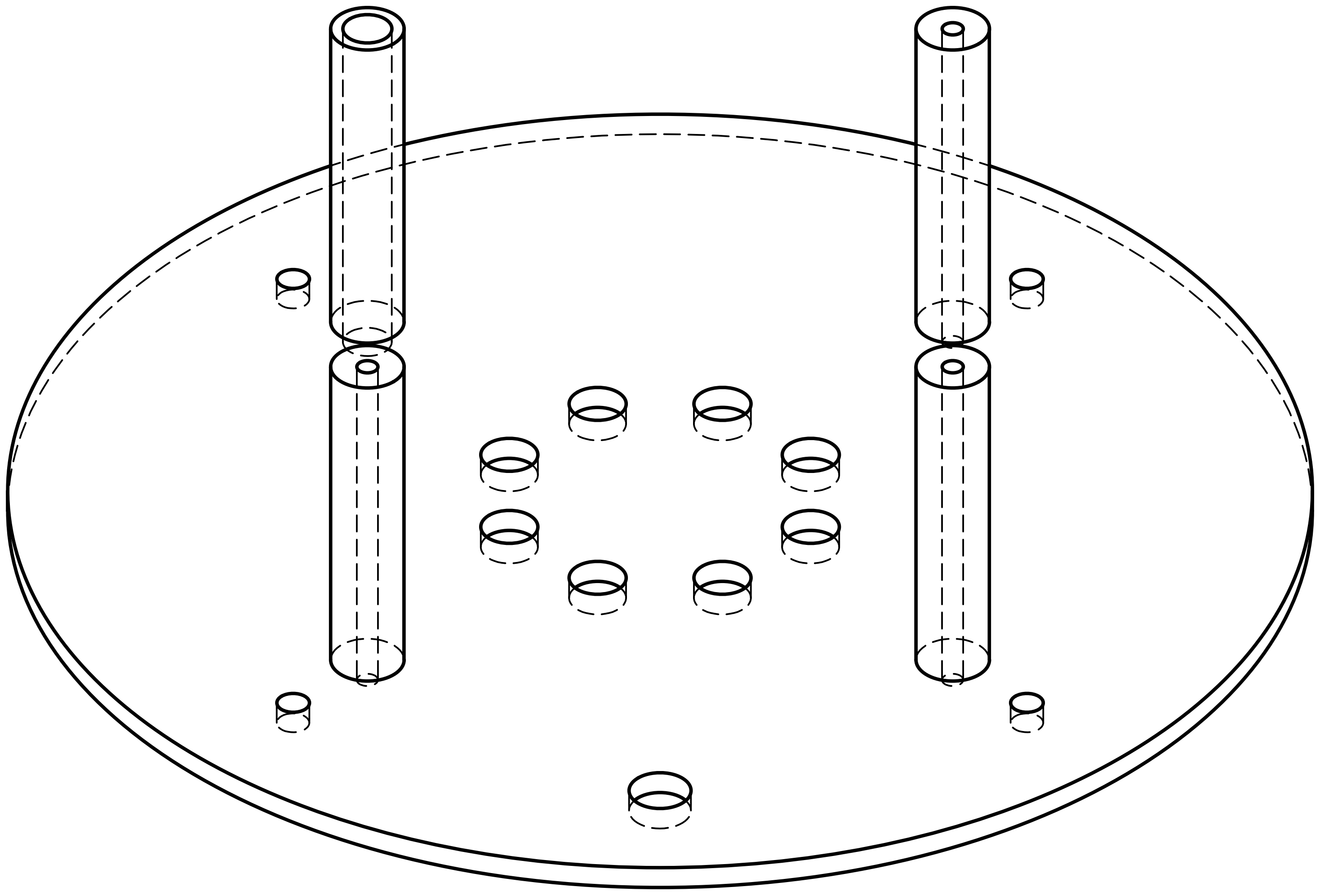

Dept.	Technical reference	Created by	Approved by
		Bunlue Kornmatitsuk and Kandit Kornmatitsuk	Bunlue Kornmatitsuk and Kandit Kornmatitsuk
		Document type	Document status
		Title: MU UV/Green LED light trap Part 3 - PCB holder	DWG No.
			Rev. / Date of issue / Sheet

Top view

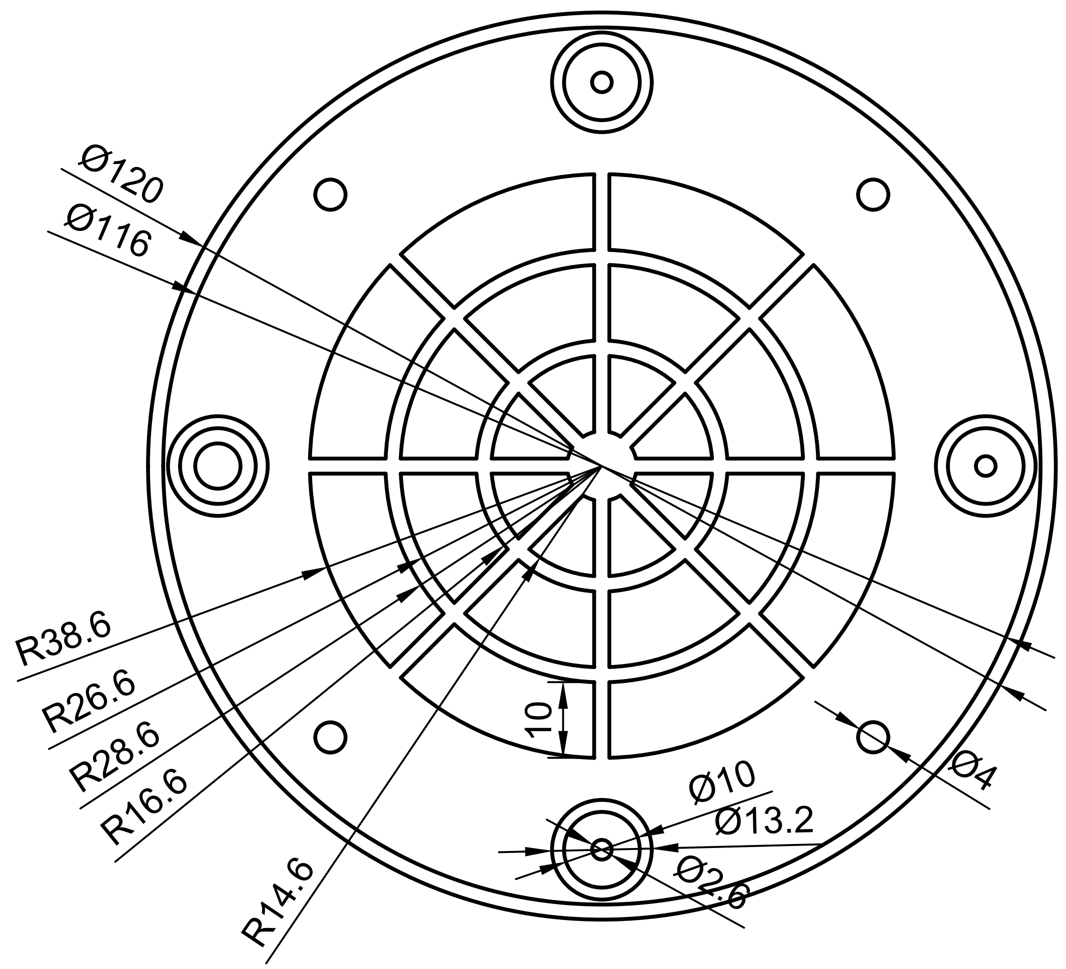

Front view

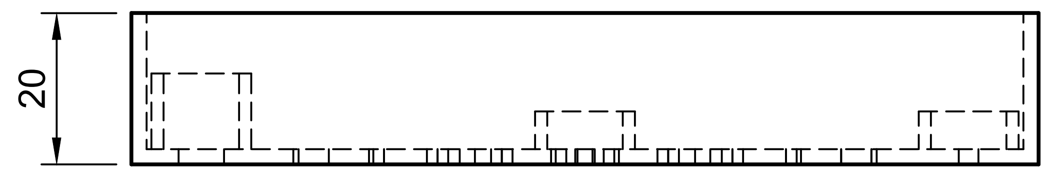

Right view

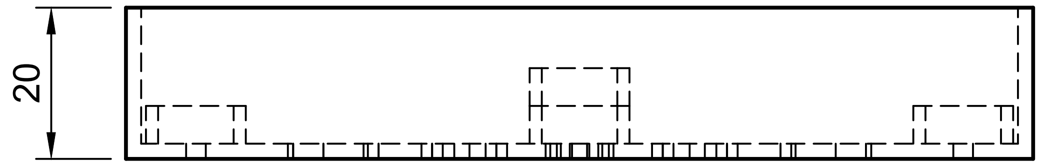

Perspective

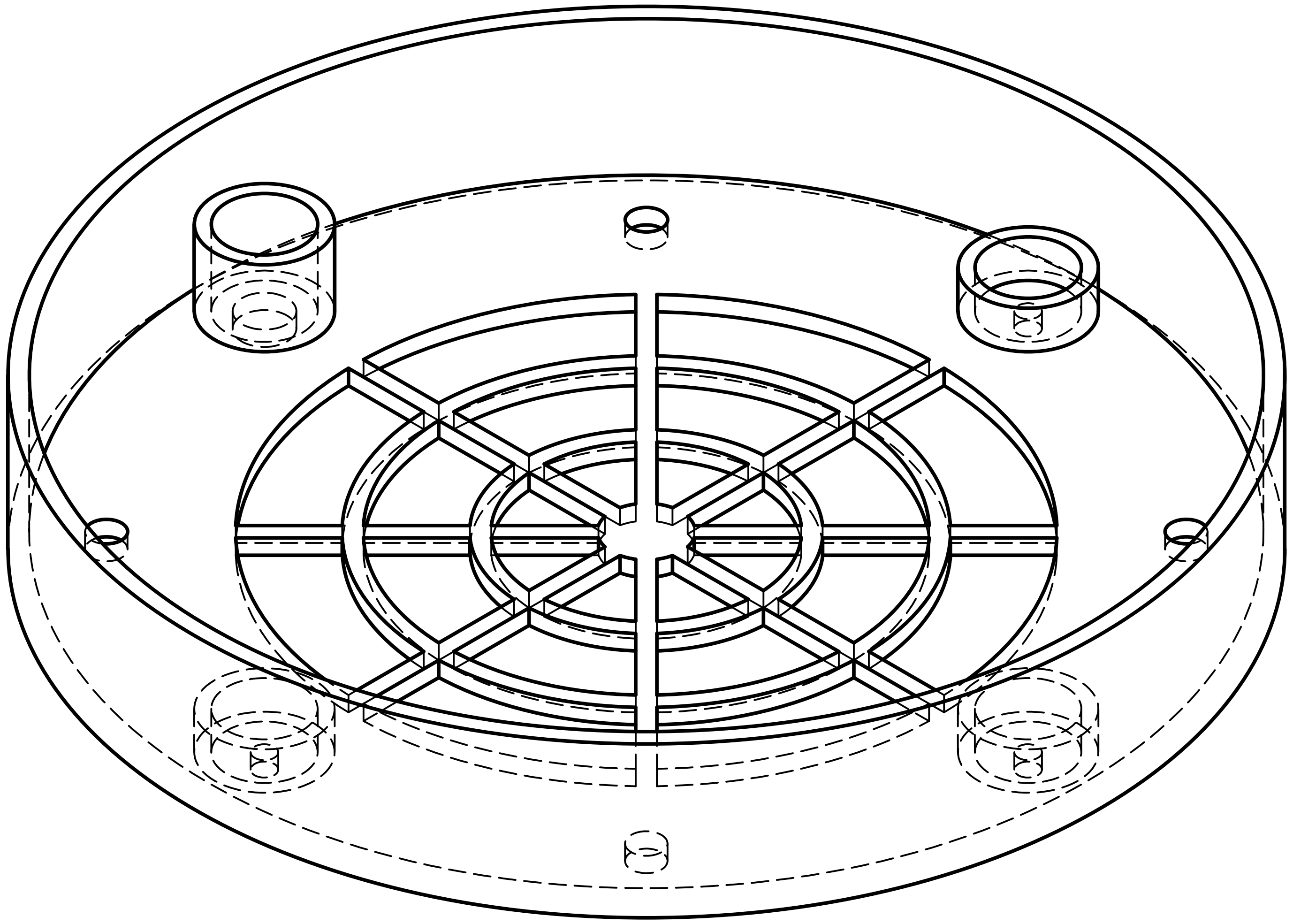

Dept.	Technical reference	Created by<br>Bunlue Kornmatitsuk and Kandit Kornmatitsuk	Approved by<br>Bunlue Kornmatitsuk and Kandit Kornmatitsuk
		Document type	Document status
		Title<br>MU UV/Green LED light trap<br>Part 4	DWG No.
			Rev. / Date of issue / Sheet

Perspective

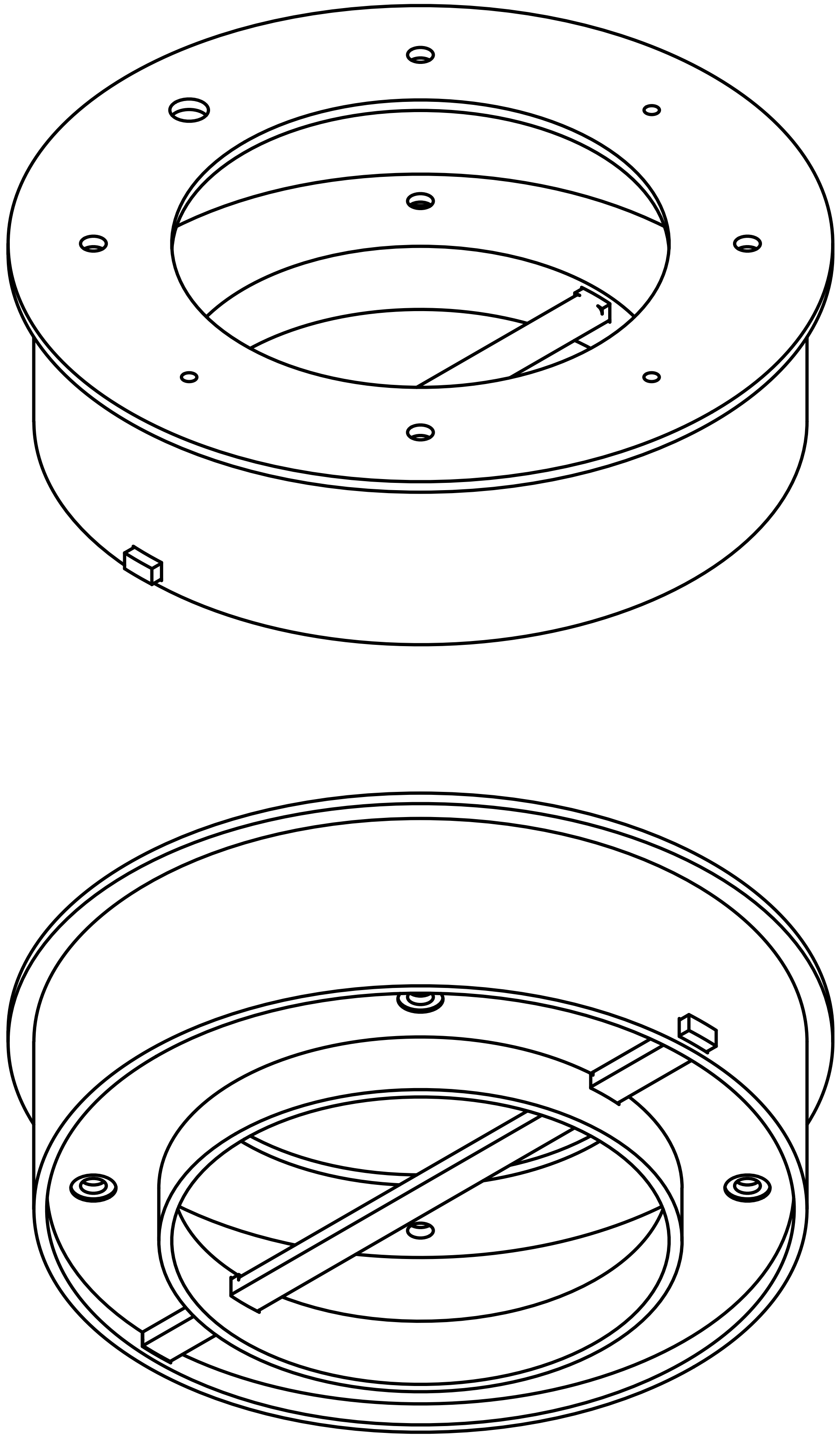

Front view

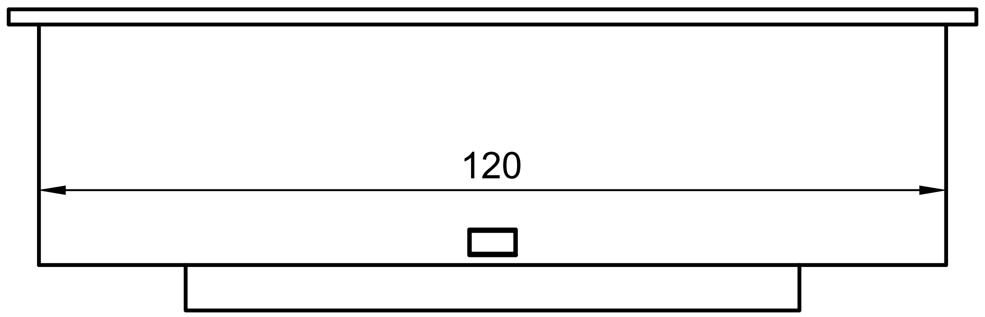

Top view

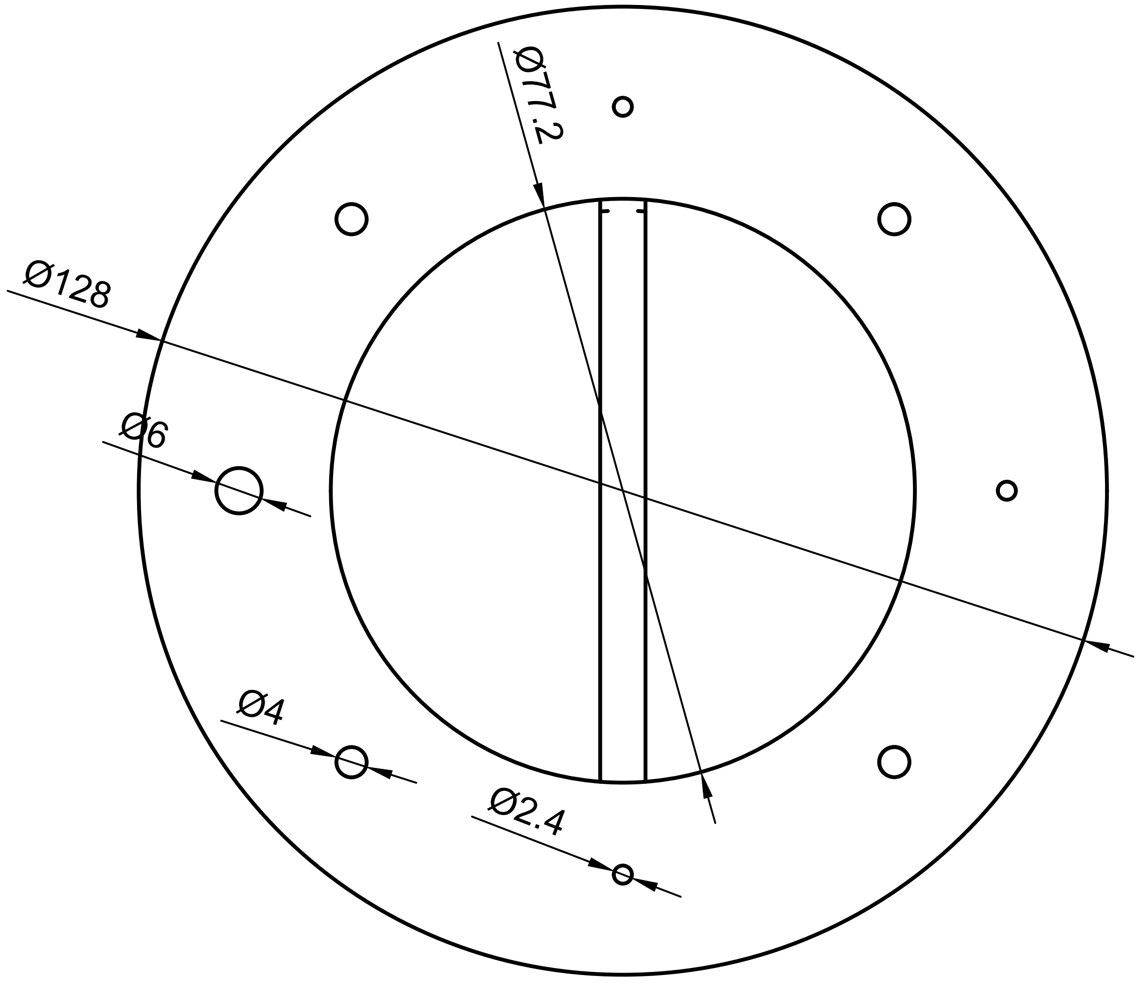

Dept.	Technical reference	Created by	Approved by
		Bunlue Kornmatitsuk and Kandit Kornmatitsuk	Bunlue Kornmatitsuk and Kandit Kornmatitsuk
		Document type	Document status
		Title: MU UV/Green LED light trap Part 5-7 - Fan housing	DWG No.
			Rev. / Date of issue / Sheet
